# Darwin’s Fancy Revised: An Updated Understanding of the Genomic Constitution of Pigeon Breeds

**DOI:** 10.1093/gbe/evaa027

**Published:** 2020-04-06

**Authors:** George Pacheco, Hein van Grouw, Michael D Shapiro, Marcus Thomas P Gilbert, Filipe Garrett Vieira

**Affiliations:** e1 Natural History Museum of Denmark, Faculty of Science, University of Copenhagen, Denmark; e2 The GLOBE Institute, Faculty of Health and Biomedical Sciences, University of Copenhagen, Denmark; e3 Bird Group, Department of Life Sciences, Natural History Museum, Tring, Hertfordshire, United Kingdom; e4 School of Biological Sciences, University of Utah; e5 NTNU University Museum, Norwegian University of Science and Technology, Trondheim, Norway

**Keywords:** pigeon breeds, genotyping-by-sequencing, population genomics, animal breeding

## Abstract

Through its long history of artificial selection, the rock pigeon (*Columba livia* Gmelin 1789) was forged into a large number of domestic breeds. The incredible amount of phenotypic diversity exhibited in these breeds has long held the fascination of scholars, particularly those interested in biological inheritance and evolution. However, exploiting them as a model system is challenging, as unlike with many other domestic species, few reliable records exist about the origins of, and relationships between, each of the breeds. Therefore, in order to broaden our understanding of the complex evolutionary relationships among pigeon breeds, we generated genome-wide data by performing the genotyping-by-sequencing (GBS) method on close to 200 domestic individuals representing over 60 breeds. We analyzed these GBS data alongside previously published whole-genome sequencing data, and this combined analysis allowed us to conduct the most extensive phylogenetic analysis of the group, including two feral pigeons and one outgroup. We improve previous phylogenies, find considerable population structure across the different breeds, and identify unreported interbreed admixture events. Despite the reduced number of loci relative to whole-genome sequencing, we demonstrate that GBS data provide sufficient analytical power to investigate intertwined evolutionary relationships, such as those that are characteristic of animal domestic breeds. Thus, we argue that future studies should consider sequencing methods akin to the GBS approach as an optimal cost-effective approach for addressing complex phylogenies.

## Introduction

Domestic animal lineages have long been appreciated for their value as model systems with which to identify the genomic mechanisms underlying their often remarkable phenotypic variation (Andersson and Georges 2004), thus contributing to our understanding of fundamental evolutionary processes (Andersson et al. 2012; Imsland et al. 2012; Rubin et al. 2012). In this regard, domestic pigeons exhibit some of the most extraordinary biological variations, and as such attracted the interest of Charles Darwin himself. Not only did he, on November 4, 1855, end a letter to his friend and colleague Charles Lyell, with the following words: “I will show you my pigeons! which is the greatest treat, in my opinion, which can be offered to human being” [sic] (Darwin 1855), but he also opted to introduce his theory of natural selection by discussing the role of artificial selection in the development of pigeon breeds (Darwin 1859). Furthermore, in his later book that focused specifically on describing the products of both animal and plant domestication (Darwin 1968), two whole chapters were dedicated to pigeons, where he expanded his rationale behind his claim that despite the immense biological diversity seen in pigeon breeds, they all descended from a single species—the rock pigeon (*Columba livia* Gmelin 1789) (Darwin 1968). It is unsurprising, therefore, that pigeons have also been of interest to geneticists since the field’s earliest days (Staples-Browne 1908; Bonhote and Smalley 1911), due to not only their astonishing phenotypic diversity but also the ease with which samples can be obtained from domestic stocks and cross-breeding experiments undertaken.

**Fig. 1.**
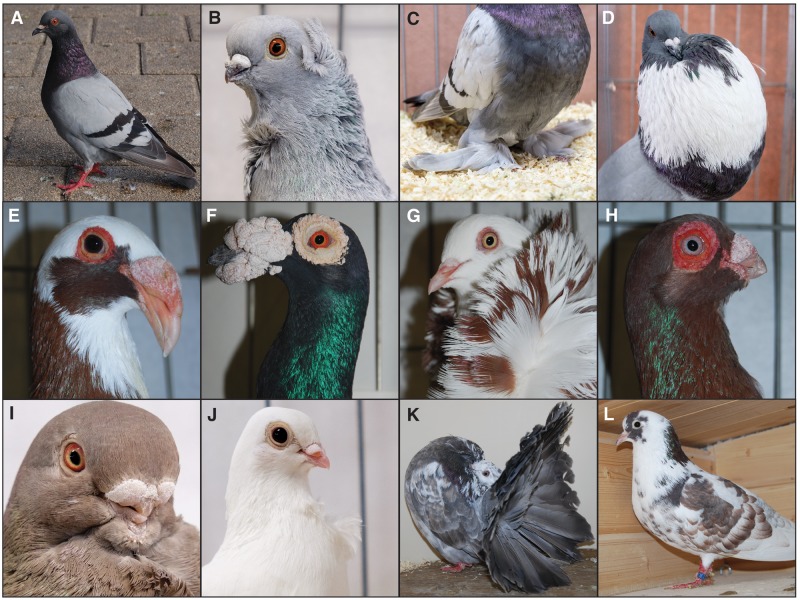
—Examples of phenotypic diversity among pigeon breeds. (*A*) Feral pigeon presenting the blue-bar ancestor morph. (*B*) Hamburg Sticken pigeon presenting the Crest, Frill, and ReducedBeak traits. (*C*) West of England Tumbler pigeon presenting the FootFeatering trait. (*D*) Pomeranian Pouter pigeon presenting the InflatedCrop trait. (*E*) Scandaroon pigeon presenting the EnlargedBeak trait. (*F*) English Carrier pigeon presenting the ProminentWattles trait. (*G*) Old Dutch Capuchine pigeon presenting the Crest trait. (*H*) Barb pigeon presenting the ProminentWattles trait. (*I*) African Owl pigeon presenting the ReducedBeak trait. (*J*) Figurita pigeon presenting the ReducedBeak and the Frill traits. (*K*) Fantail pigeon presenting the ExtraTailFeathers trait. (*L*) Laugher pigeon representing a breed that presents the SpecialVoice trait. Photos in *A*, *E–H*, *K*, and *L* were taken by H.v.G.; *B–D*, *I*, and *J* were taken by M.D.S.

It has been suggested that pigeons first came into close proximity with humans through what has been called a commensal pathway, rather than due to the deliberate action of humans (Harlan et al. 2012). Thus, even though there is considerable uncertainty concerning the precise temporal and geographic origin of the domestic pigeon (Holmes 2006; Ashley et al. 2011), it is generally thought that ancestral wild rock pigeons first began nesting in proximity to human occupations in the Mediterranean region (Hirschfeld and Tepper 2006). This was followed by the intentional construction of specialized structures to accommodate pigeons in order to facilitate the harvest of chicks and dung, and such structures were definitely in place already during the Roman era (Glover and Beaumont 1999). It is believed that the first conscious domestication efforts probably focused on traits of direct benefit to humans, such as the production of manure or meat. It was only after this initial and somewhat incipient domestication that pigeons spread with humans throughout Eurasia, and subsequently diversified under the influence of local needs, environments, and cultures. Much later, during the Victorian era, pigeon breeding for the specialized traits seen in fancy breeds became a fervent pastime, and the outcomes of pigeon breeding shifted focus to the purpose of establishing a wealth of unique breeds by the process of recombining and further developing preexisting exuberant traits. Ultimately, the complex interaction of their geographic distribution, periods of development, and purposes of selection gave rise to today’s extremely heterogeneous collection of pigeon breeds (fig. 1), which breeders have attempted to classify based on several characteristics, such as function, morphology, vocal abilities, and origin. Nonetheless, not only do these proposed classifications of pigeon breeds not follow any strict phylogenetic basis but also, unlike many other domestic animals, the breeders recorded little information about which specific breeds were crossed in order to develop the new breeds. Thus, the history of the development of pigeon breeds is much more poorly documented than for other domestic animals, such as dogs, cattle, and horses, and the paucity of literature on this subject precludes not only the formulation of a priori hypotheses but also the validation of potential findings rising from scientific studies.

In light of this lack of available information, two previous studies have attempted to reconstruct key facets of the evolutionary history of pigeon breeds given its broad interest as both a major domestic animal and its attractiveness as a model for genomic association studies (Domyan and Shapiro 2017). Although these two studies shared an overall common goal, they differed in the data they generated and analyzed. Specifically, the first analyzed a large number of breeds based on rather few genetic markers (32 unlinked microsatellites) (Stringham et al. 2012), whereas the second included considerably fewer breeds but was based on complete genomes (Shapiro et al. 2013). Considering how complex the evolutionary history of each pigeon breed is, the first was therefore limited in not being able to use large fractions of the pigeon genome to infer evolutionary history, while the second was limited by economic grounds in the number of breeds and individuals that could be studied.

Against this background, this study investigates the question of whether an intermediate approach—profiling genetic variation across a large number of unlinked loci using an economic tool such as reduced-representation library (RRLs) sequencing which enables more samples to be analyzed—would have sufficient power to reconstruct the convoluted relationships among pigeon breeds. To this end, we leveraged on the method of genotyping-by-sequencing (GBS) (Elshire et al. 2011) to generate genome-wide data for ∼200 individuals belonging to over 60 pigeon breeds. Moreover, we analyzed our newly generated GBS data alongside previously published whole-genome sequencing (WGS) data. However, because this is the first study merging GBS and WGS data sets, we decided to include in our GBS effort 23 samples that had already been sequenced through the WGS method. In this way, we were in a position to not only use the WGS data as an internal control but also assess potential limitations of GBS data.

## Materials and Methods

### Selection of Pigeon Breeds

In order to encompass as much pigeon diversity as possible and based on their worldwide popularity and morphological variety, we selected 53 recognized pigeon breeds from all 9 groups established by the National Pigeon Association of the United States of America (NPA; www.npausa.com) as well as 7 other breeds not currently recognized by the NPA (supplementary spreadsheet, Supplementary Material online). Please see the supplementary Material and Methods, Supplementary Material online, for further details on the NPA’s classification.

### Genomic Data

This study is based on the merging and analysis of two different kinds of genomic data. The first derives from three published data sets of WGS, whereas the second is a newly generated GBS data. The raw WGS data consisted of 39 purebred pigeons, 2 feral pigeons and 1 outgroup (*Columba rupestris* Pallas 1811) (Shapiro et al. 2013), 2 Pomeranian Pouters (Domyan et al. 2016), and 8 Racing Homers (Gazda et al. 2018) (supplementary spreadsheet, Supplementary Material online) as downloaded from a public database (NCBI; project numbers PRJNA167554, PRJNA284526 and PRJNA427400, respectively). Each SRA file was converted into FASTQ format (using *fastq-dump* from *SRA Toolkit v2.7*; https://github.com/ncbi/sra-tools), using default parameters plus options --split-files and --skip-technical.

### RRLs Sequencing Data

We generated GBS data for 190 samples representing 61 breeds from the collection of the Shapiro Lab at the University of Utah. Genomic DNA extractions were performed using the DNeasy Blood & Tissue Kit (Qiagen, Valencia, CA) following the manufacturer’s instructions. The extracts were quantified using the Qubit2.0 Fluorometer (Thermo Fisher Scientific, Waltham, MA). To check for molecular integrity, each DNA extract was run on a 1% agarose gel against a 1-kb ladder. Samples were sent to the Institute for Genomics Diversity – Cornell University, where the GBS method was performed following the original protocol (Elshire et al. 2011). We submitted 190 extracts that passed our filters (minimum DNA concentration of 10 ng/μl and average fragment size above 20 kb), split into two 96-well plates (PBGB_1 and PBGB_2). A negative control (water) was included in each plate in a predetermined well. At Cornell, the DNA samples were treated with the restriction enzyme *Eco*T22I before library preparation. Then, the quality of each library was inspected through the visualization of their fragment size distributions. All libraries passed quality control (appropriate concentration, fragment size distribution, and minimal adapter dimers). The respective libraries of each plate were pooled separately and then sequenced on two runs of the HiSeq 2000 apparatus (Illumina, San Diego, CA) under a protocol of single-end reads of 100 bp.

#### GBS Data Demultiplexing

We used the software *GBSX v1.3* (Herten et al. 2015) to demultiplex our GBS data allowing for one mismatch in the barcodes (-mb 1), one mismatch in the enzyme cut-site (-me 1), and ensuring that no common sequencing adapter was to be removed (-ca false).

#### Filtering for GBS Chimeric Reads

During our initial inspection of the data, we noticed that some GBS reads seemed to be chimeric. Specifically, the merging of reads derived from two or more biological cut-sites into one single artificial read (supplementary fig. 1, Supplementary Material online). We did not fully investigate these abnormal cases in the current study, but we suspect that this technical issue is caused by the undesired ligation of some cut-sites to other cut-sites during the adapter ligation step. In order to be conservative, we excluded all chimeric reads as they could bias our coverage statistics. Briefly, these were defined as those reads with 1) more than one cut-site and 2) mapped to two or more noncontiguous regions in the genome.

### WGS–GBS Samples

Among our GBS effort, we included 23 samples that had already been sequenced through the WGS method (Shapiro et al. 2013) in order to control for whether any significant bias might be introduced by our joint analyses of these two types of genomic data. Through the merging of these GBS samples with their respective WGS samples, we also created combined samples (WGS–GBS), totalling 23 triplicates.

### Read Filtering and Mapping

The software *Paleomix v1.2.5* (Schubert et al. 2014) was used to filter and map our entire data set. The two data sets (both WGS and GBS) were run with the exact same parameters (see below for details on parameters used for specific programs), except for the removal of polymerase chain reaction duplicates (that was only performed for WGS samples), and the sequencing adapters (because GBS did not use the common Illumina adapters). For the reference genome, we used a preliminary version of the Cliv_2.1 pigeon assembly (Holt et al. 2018) (https://sid.erda.dk/wsgi-bin/ls.py?share_id=ArXpW64HXt).

#### Analyzed Genomic Fraction

We restricted our analyses to only that fraction of the genome theoretically available to the GBS method. To determine this fraction, we performed an in silico digestion on the Cliv_2.1 reference assembly with the same enzyme used in our GBS protocol (*Eco*T22I) by employing *BioSeq v1.11* (Cock et al. 2009) and considered only the regions spanning 92-bp downstream and upstream each locus. Importantly, because some loci were located <92 bp apart from each other, we merged these specific loci into single locus. Hereafter, our final set of loci will be referred to as Merged_Loci.

#### Trimming of Reads

*AdapterRemoval v2.1.7* (Schubertet al. 2016) was used to filter low quality reads, trim low quality read fragments, and remove adapters using default parameters, except for a minimum read length of 30 bp (--minlength 30), collapse paired-end reads (--collapse yes), remove stretches of Ns (--trimns yes), remove consecutive stretches of bases with qualities below 15 (--trimqualities yes, --minquality 15), and discard reads with more than 40 Ns after trimming (--maxns 40).

#### Mapping

The software *BWA v0.7.15* (Li and Durbin 2009) was used to map the reads against the Cliv_2.1 reference assembly using the algorithm BWA-MEM, ignoring all reads with mapping quality below 20. Finally, to minimize increased error rates around indels, we used the software *GATK v3.6* (McKenna et al. 2010) to perform indel realignment. We used *PaleoMix* to generate mapping statistics for all loci the set Merged_Loci. Moreover, in order to explore if GBS data could be merged with WGS data without the introduction of systematic biases, we created a third replicate for each of the 23 replicated samples by merging the respective WGS and GBS BAM files; these joint samples are hereafter referred to as WGS–GBS replicates.

### Data Filtering

#### Filtering of Failed Samples

In order to filter out those samples for which a minimum number of reads were not produced, we generated a presence/absence matrix for all the loci comprised in the set of loci Merged_Loci (presence if a locus was covered by three or more reads). Due to the magnitude of the matrix, we clustered the loci (k-means with *K* = 300 clusters) and plotted the matrix as a heatmap with the samples hierarchically clustered by employing the *R* package *pheatmap* (Kolde 2012). We then inspected this heatmap (supplementary fig. 2, Supplementary Material online) by eye and decided to remove from further analyses the samples in the entire tip branch where the GBS negative controls were present.

#### Filtering of Possible Paralog Loci and Genome Portion Analyzed

We took advantage of the WGS data set to flag possible paralog loci through the conventional methods of loci exclusion based on an excess of Global Depth (GD) relative to the mean. Briefly, we first used the software *ANGSD v0.921* (Korneliussen et al. 2014) to calculate the GD per base pair of each *GBS* locus for all the WGS samples and, considering that the GD distribution follows a Poisson distribution, excluded those loci with GD considerably higher than average (>800×) (supplementary fig. 3*a*, Supplementary Material online). If not stated otherwise, this and all following plots were created using the *R* package *ggplot2 v2.2.1.9* (Wickham 2009).

### Data Analysis

We generated specific data sets to serve as inputs of the analyses conducted by performing multiple runs of data analysis using the package *ANGSD v0.921* (Korneliussen et al.2014). Although each of these runs had their own specifics, all of them obeyed some general parameters and conditions. First, only the set of loci Merged_Loci, and scaffolds longer than 1 kb (4,063 scaffolds) were analyzed, in order to avoid analyzing regions of problematic assembly (e.g., repetitive regions). Second, several filters were applied for minimum mapping quality (-minMapQ 30), minimum base quality (-minQ 20), missing data (-minInd 95%), GD (-setMaxDepth 275X per individual), minimum genotype posterior probability (-postCutoff 0.95), minimum minor allele frequency (-MinMaf 0.005), remove anomalous reads (-remove_bads 1; SAM flag above 255), adjust mapping quality for excessive mismatches (**-**C 50), perform BAQ computation (-baq 1), minimum coverage for genotype calling (-geno_minDepth 3), use *SAMtools* genotype likelihood model (-GL 1), and estimate posterior genotype probabilities assuming a uniform prior (-doPost 2). For runs where single-nucleotide polymorphism (SNP) calling was performed, we used the *ANGSD* SNP calling method (-SNP_pval 1e-6), where a Likelihood Ratio Test is used to compare between the null (maf = 0) and alternative (estimated maf) hypotheses by using a chi-square distribution with one degree of freedom.

The data sets included the following samples: data set 1 (all samples that passed our quality control plus the triplicates: 184 GBS, 50 WGS, and 23 WGS–GBS); data set 2 (all samples except for the *C. rupestris* sample that was excluded due to its high divergence: 161 GBS and 49 WGS); data set 3 (all samples except for the feral pigeon samples as well as the IndianFantail_03 and IranianTumbler_02 samples due to their uncertain origins [see supplementary Results and Discussion, Supplementary Material online]: 159 GBS and 48 WGS). Please see supplementary Materials and Methods and spreadsheet, Supplementary Material online, for details on each data set.

### Genetic Diversity

We followed the instructions provided by *ANGSD v0.921* (Korneliussen et al. 2014) to calculate the unfolded global estimate of the Site Frequency Spectrum in order to calculate the observed fraction of heterozygous sites (*H*_o_) per sample, as well as the estimates of nucleotide diversity (*π*), Watterson’s *θ* (*θ*_w_), and Tajima’s *D* (per breed) (Korneliussen et al. 2013). The observed fraction of heterozygous sites was calculated as the ratio between the number of heterozygotes and the total number of sites with information in percentage.

### Phylogenetic Reconstruction

For the Maximum-likelihood (ML) phylogenetic reconstruction, we used *RAxML-NG v0.5.1b* (Kozlov et al. 2019) to perform two phylogenetic searches using as starting topology either a Neighbor-Joining (NJ) phylogeny or 20 random topologies. The NJ phylogenetic reconstruction was based on a pairwise genetic distances matrix calculated directly from the genotype likelihoods outputted by *ANGSD* using the software *ngsDist v1.0.2* (Vieira et al. 2016) with pairwise deletion (--pairwise_del) and inferred using the software *FastME v2.1.5* (Lefort et al. 2015) with the SPR tree topology improvement (-s). Both these searches employed the GTR model with discrete GAMMA with four categories, mean category rates and ML estimate of alpha (--model GTR+G), as well as used the site repeats optimization option (--site-repeats on). We chose the phylogeny with the highest likelihood (the one starting from the NJ phylogeny) and used *RAxML-NG* to calculate bootstrap values using the bootstrap option based on 100 replicates (--bs-trees 100) and the same setup model used to compute the main phylogeny. The final bootstrapped phylogeny was visualized and plotted using the online software *iTOL v4.0.3* (Letunic and Bork 2016). The *C. rupestris* sample was used as an outgroup.

### Multidimensional Scaling

We calculated a pairwise genetic distances matrix in the same aforementioned way and used it to conduct a Multidimensional Scaling (MDS) analysis using the *R* package *cmdscale*.

### Estimation of Individual Ancestries

The software *ngsAdmix v32* (Skotte et al. 2013) was used to estimate proportions of individual ancestries for *K* = 2 up to *K* = 20 in 100 replicates using default parameters, except for tolerance for convergence (-tol 1e-6), log likelihood difference in 50 iterations (-tolLike50 1e-3), and maximum number of expectation–maximization iterations (-maxiter 10000).

### Inference of Migration Events

We ran *TreeMix v1.13* (Pickrell and Pritchard 2012) using default parameters, except for the size of block for estimation of covariance matrix (-k 100), sample size correction (-noss), round of global rearrangements after adding all populations (-global), and setting the Crupestris_01-WGS samples as the outgroup (-root Crupestris_01-WGS). Migration edges were added until residuals did not appreciably decrease (five in our case). The results were plotted using the R function plotting_funcs provided by *TreeMix*.

## Results and Discussion

### Phylogenetic Relationships among Pigeon Breeds

**Fig. 2.**
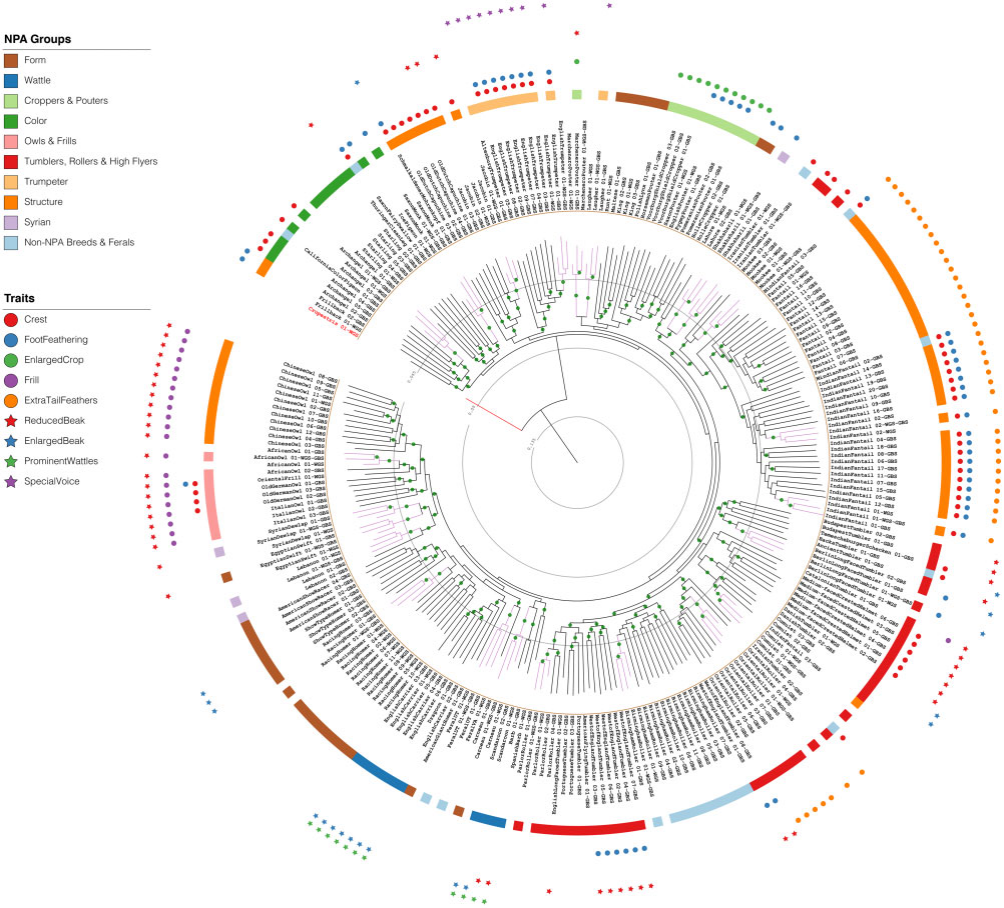
—ML phylogeny of pigeon breeds. Phylogenetic reconstruction of the relationships among over 200 pigeon individuals representing 67 breeds and 2 feral pigeons. The outgroup (*Columba rupestris*) is depicted in red, whereas the 23 triplicates are highlighted in purple. The colored ring depicts the NPA group of each sample, whereas the outermost circles and stars represent the traits present in each breed (information is given just for one sample of each triplicate). Nodes with bootstrap values above 70% are marked with green circles. The inner circular lines represent the inverted scale.

To explore whether GBS data have the power to improve our understanding of the phylogenetic affinities among pigeon breeds, and thus provide the basis for reconstructing their origins, we first conducted a ML phylogenetic analysis using data set 1. Overall, the topology of our ML phylogeny (fig. 2) is consistent with previous analyses of both WGS (Shapiro et al. 2013) and microsatellite (Stringham et al. 2012) data sets and successfully recapitulates the seven principal clades described in the latter, while also highlighting that some of the NPA groups are not monophyletic.

Our phylogeny did, however, reveal several differences concerning the topological placement of some breeds. To name a few examples, the previous analysis of WGS (Shapiro et al. 2013) placed the Jacobin as sister to the Danish Tumbler (together with the remaining TRHF), whereas we find that the Jacobin forms a clade with the Old Dutch Capuchine, which is a sister group to the Trumpeters. This Jacobin-Old Dutch Capuchine relationship has been previously reported (Stringham et al. 2012) and is consistent with their morphological resemblance (e.g., both have a well-developed hood) and shared ancestry (Moore 1735; Levi 1986; Stringham et al. 2012). Thus, we hypothesize that the affinity of the Jacobin to Tumblers and Trumpeters may be due to shared genetic background between the three groups. Another difference is that although a previous study (Shapiro et al. 2013) placed the Carneau in the clade of Pouters and other large-bodied breeds such as the Runt and King, our phylogeny places it as sister to the Scandaroon, sharing common ancestry with the Homers and the English Carrier. Originally, the Carneau was bred in France for meat production (Levi 1986). Thus, it seems logical that it could have been developed out of larger breeds, such as the early archetype Carrier (also known as Bagadet) (Moore 1735), which was probably an ancestor of the English Carrier, Scandaroon, and Racing Homers. The French Carneau was later imported to the United States around 1900, where its appearance has been dramatically modified through outcrossing with other breeds, and the American version has today a much larger size when compared with the French version; inasmuch as the modern day American Carneau might be deemed a different breed (Levi 1986). Therefore, we believe that a breed such as Carneau that resulted from recent hybridization among different breeds might be expected to group with more than one breed group in different analyses with different data sets because the placement of a hybrid breed on a bifurcating phylogeny is prone to vary.

We reason that these phylogenetic discrepancies might have been caused by differences in sample size, quality of the data, and phylogenetic methods used in the previous studies. Specifically, the phylogenetic analysis performed by Shapiro et al. (2013) analyzed a lower number of samples (41), included linked loci (no Linkage Disequilibrium [LD] pruning was performed) and employed a simpler phylogenetic method (NJ reconstruction based on a presence/absence matrix). On the other hand, the phylogeny based on microsatellites (Stringham et al. 2012) was based both on a lower number of breeds and on loci (40 and 32, respectively). Although acknowledging methodological differences, we believe that our phylogeny is the most informative and relevant seeing that it includes a higher number of breeds and was computed through a robust phylogenetic method.

We also noted how several pigeons labeled as belonging to a single breed were found on different branches of our phylogeny. For example, the two Mindian Fantail (a breed not recognized by the NPA) samples did not form a monophyletic group. One sample is an outgroup to all Indian Fantails, whereas the other is an outgroup to all Fantails and Indian Fantails. We note that the Mindian Fantail is the product of a recent outcross, developed with the goal of miniaturizing the Indian Fantail. In order to achieve this result, breeders outcrossed the Indian Fantail with other breeds (namely small Tumblers; D. Skiles, personal communication to M.D.S.), which could explain these phylogenetic incongruences. Similarly, a recent study found that dog breeds under development would also have a tendency to not form monophyletic clades (Parker et al. 2017). Moreover, the two samples belonging to the Iranian Tumbler breed (a breed also not recognized by the NPA) did not cluster together either. This may indicate that one of these samples was recently outcrossed or erroneously labeled.

Overall, although our results highlight that there is a general phylogenetic rationale behind the NPA classification, some considerable discrepancies are obvious. The Form group is clearly not monophyletic, something that is not unexpected given that this group is defined based on selection toward a specific body form, and the breeds included in this group have very distinct origins (e.g., heavy breeds originally developed for meat production and breeds originally selected for an improved homing performance). Despite its small number of breeds, the Wattle group is also not monophyletic, as the English Carrier and Dragoon cluster together with breeds derived from the Racing Homer. However, we do not find this surprising because some of the ancestors of these Wattle breeds were used in the development of the modern Racing Homers (Tegetmeier 1871; Stringham et al. 2012; Shapiro et al. 2013). While the Croppers and Pouters group form an almost monophyletic group as they are only rendered polyphyletic by the Marchenero Pouter. Even though Croppers and Pouters are generally similar, Spanish pouters are morphologically distinct and inflate their crops differently from the other Croppers and Pouters. Moreover, Spanish pouters are used for thieving, which adds a premium on flying ability. Therefore, it is reasonable to believe that the genomic background of Spanish pouters is somewhat different from those of other pouters, which might have led to some level of phylogenetic inconsistency on a bifurcating phylogeny. The Colour group is also paraphyletic as it includes the Frillback breed, which is considered a Structure breed by the NPA. The Owls and Frills group is almost monophyletic, made paraphyletic by the classification of the Chinese Owl as a Structure breed. Despite being the largest group, the TRHF group is mostly monophyletic, with the exception of the Mookee breed that is an outgroup to the Fantails (Structure). This pattern is not surprising, because the Mookee and Fantail breeds were believed to be closely related as these breeds used to be known as the Narrow and Broad Tail Shaker, respectively (Moore 1735; Sell 2009). The Trumpeter group is not monophyletic, possibly indicating that the Laugher derived voice is analogous to that found in Trumpeters, which would be in accordance with the diversity of this trait as different breeds in this group show different kinds of voices (e.g., drumming and laughing voices) (Marks 1975). Because the Structure group includes breeds with different genetic affinities, it is unsurprising that this group is made polyphyletic through inclusion of breeds that show close phylogenetic relationships with other groups, such as the Old Dutch Capuchine, which holds phylogenetic relationship with the Trumpeters. Finally, the Syrian group is paraphyletic as it includes the Egyptian Swift, which is considered a Form breed by the NPA.

Pigeon breeds are known for their variety of phenotypic traits (fig. 1), but it is not always clear when and how many times these traits emerged. Our expanded phylogeny (fig. 2) widens our understanding of the evolution of these derived traits and, in agreement with previous phylogenetic analyses (Stringham et al. 2012; Shapiro et al. 2013), shows that some are apomorphic, whereas others are spread across the entire phylogeny. Specifically, it shows that the traits Crest, FootFeathering, ExtraTailFeathers, ReducedBeak, and EnlargedBeak are scattered across breeds which belong to several NPA groups. This trait distribution pattern indicates that either these traits independently arose multiple times during the evolution of pigeon breeds or were transferred across different groups by intentional and careful breeding. On the other hand, the trait EnlargedCrop is only found in breeds belonging to the Croppers and Pouters group and these are restricted to one section of the phylogeny; the Frill trait is limited to breeds belonging to the Structure and Owls and Frills groups, however these breeds cluster together in the phylogeny; the ProminentWattles trait is confined to breeds belonging to the Wattle group and these are found in a single portion of the phylogeny; and the SpecialVoice trait is unique to breeds belonging to the Trumpeter group, which form a single cluster except for the Laugher. This trait distribution pattern supports the idea that these traits were probably only developed once throughout the history of pigeon breeding. This updated knowledge of the phylogenetic distribution of these derived traits might, in some cases, help future investigations attempting to reveal the genomic underpinnings behind the astonishing biological diversity seen in pigeon breeds (Domyan and Shapiro 2017).

All in all, the GBS method yielded sufficient analytical power to elucidate the overall phylogenetic affinities among pigeon breeds. Thus, we believe that RRLs sequencing approaches might represent the best cost-benefit tradeoff currently available for studies seeking to reveal complex evolutionary relationships, such as those that are characteristic of animal domestic breeds.

### Genetic Variability of Pigeon Breeds

Domestic lineages usually have complex evolutionary histories shaped by strong artificial selection, population bottlenecks, and periods of inbreeding that are occasionally punctuated by admixture among lines (Makino et al. 2018). These evolutionary forces affect the levels of genetic diversity of each developed breed differently, and these genomic fingerprints can be used to shed light on past evolutionary processes. Thus, we took advantage of the fact that we have several samples for some of the studied breeds and calculated the observed levels of heterozygosity (*H*_o_), nucleotide diversity (*π*), Watterson’s *θ* (*θ*_w_), and Tajima’s *D* across the pigeon genome.

Values of *H*_o_ were calculated for all samples in data set 1, except IndianFantail_03 and IranianTumbler_02, due to their inconsistent phylogenetic placement (see supplementary Results and Discussion, Supplementary Material online). All other statistics were calculated for the 12 breeds that had 5 or more individuals, as these genetic estimates only apply for population data (for the 23 triplicates only the WGS libraries were used). The individual *H*_o_ levels among the pigeon breeds ranged from 0.0679% to 0.2395% (mean 0.1571%), with considerable variation within each breed and the presence of several outliers (supplementary fig. 5, Supplementary Material online). In general, these values are similar to those reported for seven duck breeds (mean 0.1530%) but are lower than those reported for two wild populations of mallard (mean 0.3009%) (Zhang et al. 2018) and are consistent with what would be expected of a lineage that has been subject to the evolutionary forces imposed by the domestication process (Groeneveld et al. 2010; Makino et al. 2018).

The mean values of *π* ranged from 0.0016 to 0.0027 (mean 0.0021) (supplementary fig. 6*a*, Supplementary Material online), whereas the *θ*_w_ values ranged from 0.0013 to 0.0026 (mean 0.0019) (supplementary fig. 6*b*, Supplementary Material online). Our estimates of *π* are similar to those other of domesticated avian breeds (0.0020–0.0028) (Zhang et al. 2018) but considerably lower than those calculated for wild counterparts of domesticated avian species, such as the Mallard (∼0.0040) (Zhang et al. 2018) and the Red Junglefowl (0.0052) (Lawal et al. 2018), as would be expected considering the long history of extensive artificial selection experienced by the domestic lineages. Previously reported values for pigeon breeds (0.0036) (Shapiro et al. 2013) are higher than our estimates, but this might be due to the fact that that data set included resequenced genomes of both domestic and feral pigeons.

Next, to test for evidence of rapid population contraction (bottlenecks), we calculated Tajima’s *D* for each breed. Estimates ranged from 0.1752 to 0.8428 (mean 0.4654) (supplementary fig. 6*c*, Supplementary Material online), in accordance with reports for purebred lineages of other domestic animals such as quail (Wu et al. 2018) and sheep (Pan et al. 2018). These positive values probably reflect the recurrent history of bottlenecks inherent in the domestication process. Moreover, these values show a negative correlation with *H*_o_ (Pearson correlation = −0.5128974; *P* value = 0.08815), in agreement with lower variability during the bottleneck. The only exceptions are the English Carrier and Oriental Roller, which show relatively low values of both statistics. When these breeds are excluded, the correlation becomes stronger and highly significant (Pearson correlation = −0.859119; *P* value = 0.001449). This could indicate that these breeds did not go through a very strong bottleneck during domestication but have been kept relatively isolated ever since. Interestingly, the Fantail, Indian Fantail, and Chinese Owl breeds showed the highest Tajima’s *D* and lowest genetic diversity according to *θ*_w_, suggesting that these breeds underwent a severe bottleneck.

Interestingly, our results also showed that the Archangel and Starling breeds (both belonging to the Colour group) had the highest levels of genetic diversity. These breeds show a wide range of colors and plumage patterns, which are often maintained as somewhat closed lines. Since our samples came from individuals with different phenotypes (data not shown), they probably represent different lineages within each breed. Therefore, the observed high level of genetic diversity might be an artifact due to the presence of some level of genetic structure within these breeds. The Racing Homer also showed high genetic diversity, but we believe that this could be explained by 1) its much larger effective population size given that it is raised in large numbers across the globe and 2) it is a relatively young breed (∼200 years old) that was developed from many different breeds (Tegetmeier 1871).

Taken together, these results demonstrate that domestic pigeons adhere to the main trend of domestic lineages, showing reduced levels of genetic diversity probably originated from a recurrent history of population bottlenecks. Furthermore, likely due to variations in domestication periods, geographical origins, and domestication purposes, these results also indicate that artificial selection has imprinted different pigeon breeds with distinct genomic signatures.

### Population Structure across Pigeon Breeds

Continued artificial selection on domestic lineages commonly leads to pronounced population structure, chiefly among established breeds and lines (e.g., Alves et al. 2015; Signer-Hasler et al. 2017). Although previous studies have investigated the patterns of population structure among pigeon breeds, they examined either fewer genetic loci (32 microsatellites) (Stringham et al. 2012) or breeds (37 breeds) (Shapiro et al. 2013). As it has been demonstrated that genome-wide SNPs tend to better recapitulate evolutionary relationships in comparison with microsatellites (Väli et al. 2008; Gärke et al. 2012; Fischer et al. 2017), we used our larger data set to unravel at a finer scale the patterns of population structure among pigeon breeds through the analyses of Proportions of Individual Ancestries (Admixture) and MDS performed using data set 2.

**Fig. 3.**
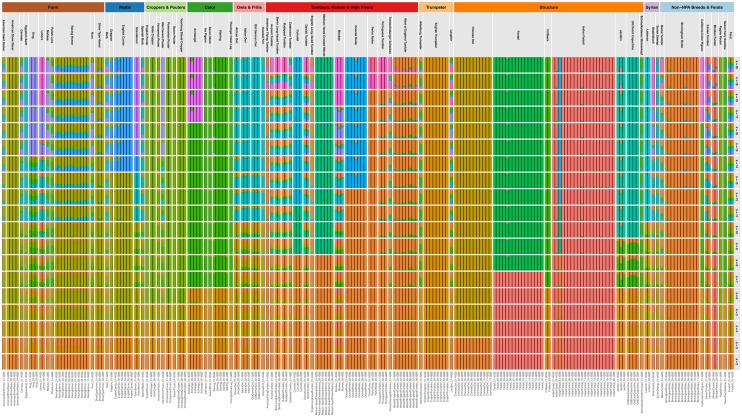
—Admixture proportions of pigeon breeds. Individuals are represented by columns, whereas rows depict the Admixture proportions based on the assumption of different numbers of ancestral populations (*K* = 2–20). Individuals are sorted by breeds (gray upper labels) and grouped per NPA groups (colored upper labels; the colors used are as in the phylogeny presented in fig. 2).

**Fig. 4.**
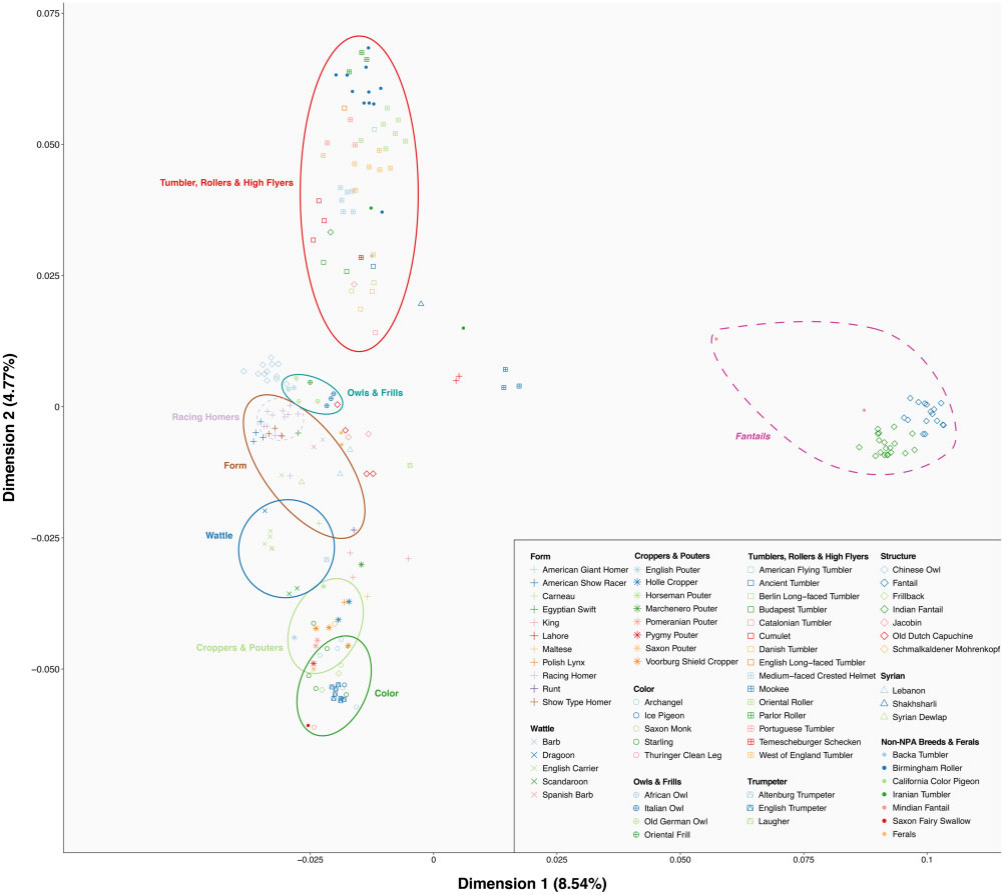
—MDS analysis of pigeon breeds. Dimensions 1 and 2 are plotted and each point on the plot represents a single individual. The colored solid ellipses represent the rough distribution of the most homogeneous NPA groups (the colors used are as in the phylogeny and Admixture plots), whereas the dashed ellipse depicts the distribution of the Fantail breeds.

Our Admixture (fig. 3) and MDS (fig. 4) analyses show considerable population structure among pigeon breeds, consistent with the findings of previous studies (Stringham et al. 2012; Shapiro et al. 2013). This likely arose as a direct product of continuous artificial selection. It is worth noting that the Jacobin appears midway between Tumblers and Trumpeters on the MDS, further supporting its shared ancestry with both these groups. The Laugher does not share ancestry with Trumpeters and is placed next to the Structure group on the MDS plot, further supporting the hypothesis that the Laugher derived voice is analogous to that found in Trumpeters (see above) (Marks 1975).

These analyses also provide extra evidence that some NPA groups seem to be relatively homogeneous and genetically isolated, such as the TRHF, the Owls and Frills, the Form, and the Wattle breeds. On the other hand, other groups are more genetically similar, such as the Croppers and Pouters and the Colour breeds. The latter appears to include the English Trumpeter, but this breed is separated on the MDS dimension 3 (supplementary fig. 8, Supplementary Material online). As also found by a previous study (Gazda et al. 2018), we highlight that a well-structured cluster is formed by the Racing Homers, despite the fact that we included samples from both Europe and North America. The Racing Homer breed was first established in Europe (Levi 1996), and our results indicate that descendant populations in North America remained genetically similar. Furthermore, also being consistent with previous results (Stringham et al. 2012), both feral samples had the greatest number of ancestry components at *K* = 20, as might be expected from an admixed feral population (Wang et al. 2017).

### Inference of Admixture Events

The evolutionary history of pigeon breeds is rife with interbreed crosses. Traditional population genetic analyses attempt to infer relationships among populations as a bifurcating phylogeny. However, simple bifurcating phylogenies may not correctly represent population histories (Cavalli-Sforza and Piazza 1975; Pickrell and Pritchard 2012). Thus, in an attempt to detect past admixture events among pigeon breeds, we conducted using data set 3 a phylogenetic analysis employing a method that fits a population graph (allowing for both population splits and mixtures) to the allele frequency correlation patterns among a set of the sampled populations (Pickrell and Pritchard 2012).

**Fig. 5.**
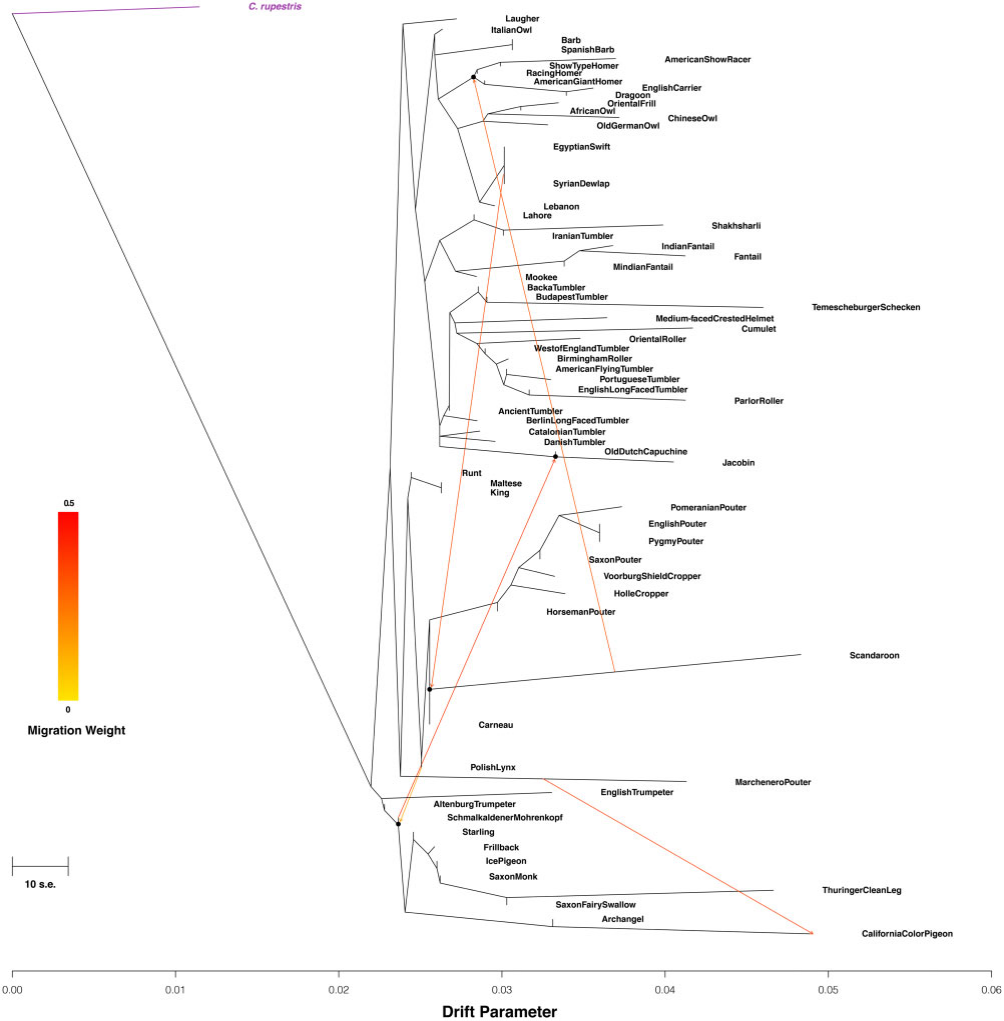
—TreeMix ML phylogeny of pigeon breeds. Five migration events among different pigeon breeds are represented by arrows on the phylogenetic graph. The scale bar indicates ten times the average S.E. The outgroup is marked in purple. The model residuals are plotted in supplementary figure 9, Supplementary Material online.

We found overall congruence between the ML (fig. 2) and TreeMix phylogenies when no hybridization events were allowed (supplementary fig. 9*a*, Supplementary Material online). Although many more hybridization events would be expected to have occurred during the development of fancy breeds, we decided to restrict our analysis to the hybridization events that occurred during the evolution of pigeon breeds that exhibit the five strongest signatures (fig. 5). The first hybridization event is from the Schmalkaldener Mohrenkopf to the node joining the Jacobin and the Old Dutch Capuchine (that now appears next to the Danish Tumbler, as previously seen [Shapiro et al. 2013]). The Jacobin is recognized as an ancient breed, and it was used to improve the feather length of the Schmalkaldener Mohrenkopf (personal communication from breeders to H.v.G.). Thus, we believe that the genomic affinity between these two breeds seen in our study as well as in a previous one (Stringham et al. 2012) might well explain this hybridization event. The second migration is from the Scandaroon to the node encompassing all Homers, the American Show Racer, the English Carrier, and the Dragoon. The English Carrier is considered to be closely related to the Scandaroon and, given that both breeds share common ancestry with the breeds that were used in the development of the Homers (Levi 1986), this is not unexpected (Levi 1965). The third migration is from the Syrian Dewlap to the node joining the Carneau and Scandaroon. Despite their lack of morphological similarity, these are thought to have originated (or have ancestors) in neighboring regions in the Middle East (Moebes 1950). Thus, we believe that this migration could be due to a deep relationship relating to a common founder population. The fourth migration is from the Polish Lynx to the node shared by all the Colour pigeons. The Polish Lynx is known to be derived from a Field Pigeon (Colour group) and a Cropper and Pouter pigeon (Schütte et al. 1971; Marks 1975), which could explain this migration. The fifth and final migration happens from the Marchenero Pouter to the California Colour Pigeon. The latter breed was developed very recently and is yet to be recognized by the NPA. However, its creator (Frank Mosca) attests that the Marchenero Pouter was used in the development of the California Colour Pigeon (www.angelfire.com/ga3/pigeongenetics/ccpstandard.html). Given that the last two admixture events are well known and described, this provides an additional measure of confidence about the reliability of the older admixture events we detected.

In summary, we found that admixture events underlying the development of pigeon breeds can be reconstructed with genomic data. Thus, as more genomic data sets are generated, we expect to learn much more about the history of many other pigeon breeds.

## Conclusion

Being a product of continuous artificial selection over several centuries, the NPA today recognizes ∼230 pigeon breeds, whose phenotypes exhibit incredible diversity. With the ultimate goal of further improving the current phylogenetic knowledge on pigeon breeds, we conducted the most inclusive genomic study to date for the group, considerably improving our understanding of the complex evolutionary affinities among pigeon breeds. We also demonstrate that there is considerable population structure across pigeon breeds as a result of intensive artificial selection, and that pigeon breeds can indeed be classified into distinct groups with different levels of genetic homogeneity and evolutionary histories. In this regard, the current NPA classification has some phylogenetic sense, even though it was not intentionally developed based on phylogenetic aspects. Furthermore, our results corroborate previous studies which showed that although some derived traits present in pigeon breeds were probably inherited from a common ancestral breed, others are distributed across the phylogeny (probably due to intentional transfer of traits from one breed to another) (Shapiro et al. 2013; Domyan et al. 2014, 2016; Vickrey et al. 2018). Nonetheless, because the majority of the pigeons analyzed in our study were collected outside the regions where their respective breeds were initially established, our results should be interpreted with caution because these breeds might have been considerably altered once exported from their place of origin (Parker et al. 2017). Thus, we advocate that future genomic studies on breeds should strive to sample individuals within the breeds’ respective regions of origin. Despite this potential sampling caveat, we believe that our study is an important step toward our better comprehension of the evolutionary affinities among pigeon breeds, comprehension which is indispensable for the elevation of the domestic pigeon as a model organism for genomic investigations (Domyan and Shapiro 2017).

Because we also demonstrate that GBS data are sufficient for most phylogenetic and population genetic analyses despite minor biases (see supplementary Results and Discussion, Supplementary Material online), our findings encourage us to believe that applying the GBS or similar RRLs sequencing methods across the full range of recognized pigeon breeds would be a milestone toward this specific goal. Nonetheless, seeing that sometimes the main goal in pigeon research is the ultimate identification of the genomic substrate of selected traits (Shapiro et al. 2013; Domyan et al. 2016; Domyan and Shapiro 2017; Vickrey et al. 2018; Boer et al. 2019), and given the high levels of LD in pigeons (supplementary fig. 10, Supplementary Material online), we also tried to investigate whether GBS data could be used for GWAS (supplementary fig. 11, Supplementary Material online). Our results indicate that GBS data do not yield enough resolution for this kind of analysis (see supplementary Results and Discussion, Supplementary Material online). Therefore, considering the continuous advancements in sequencing technologies that have been making WGS more and more affordable, we reason that investigations in several branches of the biological sciences where the pigeon has been used as a model system would benefit from the generation of more full genomes, ideally even for all the recognized pigeon breeds. More specifically, it would undoubtedly facilitate the proliferation of comparative genomics studies taking advantage of the entire assortment of biological features seen in the group, as has been performed for other domestic animals (Axelsson et al. 2013; Imsland et al. 2016; Carneiro et al. 2017; Alberto et al. 2018). Furthermore, we believe that the pigeon’s short generation time, easy animal handling, and relatively small genome compared with other model organisms place this group at the privileged position for scientific queries as was foreseen by Charles Darwin more than 150 years ago.

## Supplementary Material

Supplementary data are available at *Genome Biology and Evolution* online.

## Supplementary Material

evaa027_Supplementary_Data

## Notes

**Data deposition:** This project has been deposited at Sequence Read Archive under the accession PRJNA485426.
