# Supplementary material for: Darwin’s Fancy Revised: An Updated Understanding of the Genomic Constitution of Pigeon Breeds
Source: Genome Biol Evol. 2020 Apr 6;12(3):136–50. doi: 10.1093/gbe/evaa027 (PMC7144551; doi:10.1093/gbe/evaa027)
Supplement: evaa027_Supplementary_Data [file evaa027_supplementary_data.zip › PBGP--SI_RS.pdf]

SUPPLEMENTARY INFORMATION

Darwin's Fancy Revised: An Updated Understanding of the Genomic Constitution of Pigeon Breeds

George Pacheco[1,2*], Hein van Grouw[3], Michael D. Shapiro[4], M. Thomas P. Gilbert[1,2,5] & Filipe Garrett Vieira[1]

[1]Natural History Museum of Denmark, Faculty of Science, University of Copenhagen, Øster Voldgade 5–7, 1350 Copenhagen, Denmark.
[2]The GLOBE Institute, Faculty of Health and Biomedical Sciences, University of Copenhagen, Øster Farimagsgade 5A, Copenhagen, Denmark.
[3]Bird Group, Department of Life Sciences, Natural History Museum, Akeman Street, Tring, Herts. HP23 6AP, United Kingdom.
[4]School of Biological Sciences, University of Utah, 257 South 1400 East, Salt Lake City, UT 84112, USA.
[5]NTNU University Museum, Norwegian University of Science and Technology, NO-7491, Trondheim, Norway.
*george.pacheco@snm.ku.dk

Table of Contents

Supplementary Materials & Methods 2
Selection of Pigeon Breeds 2
Sequencing Output & Data Processing 2
Molecular Sexing 2
Dataset Specificities 2
Linkage Disequilibrium 3
Sequencing Output & Data Processing 3

Supplementary Results & Discussion 4
Sequencing Output & Data Filtering 4
Molecular Sexing Based on GBS Data 5
Assessment of the Distribution of GBS Sites Across the Pigeon Genome 5
WGS / GBS Samples 5
Detailed Phylogenetic Description 6
Within/Between Genetic Distances of Pigeon Breeds 6
Abnormal Samples 7
Notes on the NPA Classification 7
Linkage Disequilibrium Levels Across Pigeon Breeds 7
Assessing GBS Performance for Genome-wideAssociation Studies 7

Supplementary References 9

Supplementary Figures 10

Supplementary Fig. 1. Schematic example of a GBS chimeric read. 10

Supplementary Fig. 2. Heatmap based on the presence/absence matrix. **11**
Supplementary Fig. 3. Global Depth (GD) density plots. **12**
Supplementary Fig. 4. Violin plot of genetic distances. **13**
Supplementary Fig. 5. Proportion of observed heterozygous sites. **14**
Supplementary Fig. 6. Population genetics estimates per breed. **15**
Supplementary Fig. 7. Sites and SNPs information. **17**
Supplementary Fig. 8. Multidimensional Scaling Analysis (MDS). **18**
Supplementary Fig. 9. TreeMix phylogenies for pigeon breeds and model residuals. **19**
Supplementary Fig. 10. Linkage-disequilibrium (LD) across pigeon breeds. **25**
Supplementary Fig. 11. Manhattan plot for the GWAS analysis on the derived traits. **26**

Other Supplementary File

PBGP Supplementary Spreadsheet

Supplementary Materials & Methods

Raw Data

All the raw data are stored on the *University of Copenhagen*'s long term storage server (ERDA) and can be downloaded from https://sid.erda.dk/wsgi-bin/ls.py?share_id=BTZBGV6Tsj.

Selection of Pigeon Breeds

Although many countries have their own national pigeon associations that classify pigeon breed into specific groups, we elected to follow the classification from the National Pigeon Association of the United States of America (NPA; www.npausa.com) given its status as one of the largest associations of pigeon breeders. Thus, we used the NPA current classification as a reference for pigeon breed grouping. We did so for the sake of convenience, and acknowledge that some of these groupings are based on historical reasons and do not necessarily reflect phylogenetic relationships. The NPA publishes a book that describes and classifies all of its recognized pigeon breeds, and, according to NPA's 2010 Book of Standards (National Pigeon Association 2010), all recognized breeds are divided into nine groups, depending on their function, morphology, vocal abilities, presumed geographic origin, etc. These groups are named Form (breeds mainly selected for their body form), Wattle (relatively small group including breeds having pronounced wattles), Croppers & Pouters (breeds that present conspicuous crops), Colour (breeds chiefly developed for their colours and markings), Owls & Frills (breeds generally with a short beak and chest frill), Trumpeters (a group of breeds selected for voice characteristics), Tumblers, Rollers & High Flyers (TRHF; the largest group covering all breeds with a performing background), Structure (diverse group of breeds selected for their exuberant ornamentation) and Syrian (breeds grouped together mainly due to their assumed common geographical origin).

Sequencing Output & Data Processing

Since the first lane of sequencing on plate PBGB_1 did not produce the minimum desired number of reads (250 M), this whole plate was re-sequenced. Even after this extra sequencing on plate PBGB_1, columns 1-3 of this plate as well as of plate PBGB_2 did not produce enough reads. Thus, the samples placed in these wells were pooled together and re-sequenced once again on a separate lane. The reads of samples having reads coming from different lanes were merged together using the Lane option of the PaleoMix software (Supplementary Spreadsheet).

Although we caution that bugs might emerge due to changes in software versions, the entire pipeline for this project alongside with its documentation to serve as a guideline for the analyses herein performed can be found at: https://github.com/layka-pacheco.

Molecular Sexing

We compared the coverage between the Z sex chromosome (haploid in female birds) and an autosome chromosome of similar size (chromosome 6) for all our samples, under the assumption that in female birds approximately half the number of reads would map to the Z chromosome in comparison to the number of reads that would map an autosome chromosome of similar size. However, since the Cliv_2.1 reference assembly is only at the scaffold level, we took advantage of a chromosome level pigeon assembly (Damas et al. 2017) in order to identify which scaffolds belong to chromosomes 6 and Z. To do so, we independently blasted these two chromosomes against our reference genome using Blast+ v2.6.0 (Camacho et al. 2009), requiring a minimum percentage identity (-perc_identity 95), e-value (-evalue 1e-5), alignment length (greater than 1 kb), subject length (greater than 10 kb), and a proportion of gaps in the alignment smaller than 1%. All subject sequences with alignments that passed the filters were assumed to belong to the respective query chromosome (either 6 or Z), and their coverage were calculated.

Dataset Specificities

Dataset 1 comprised 1,997,420 total sites (including

monomorphic) with coverages at potential GBS loci ranging from 7.03X to 323.05X (mean 85.22X), and missing data from 0% to 1.55% (mean 0.16%); Dataset 2 comprised 26,082 SNPs with coverages at potential GBS loci ranging from 7.02X to 322.93X (mean 84.82X) and missing data from 0% to 1.89% (mean 0.24%); Dataset 3 comprised 26,504 SNPs with coverages at potential GBS loci ranging from 7.01X to 321.60X (mean 84.95X) and missing data from 0% to 1.94% (mean 0.24%) for genotype likelihoods and from 0.03% to 21.83% (mean 1.74%) for genotype calling.

Linkage Disequilibrium

We used the software ngsLD (https://github.com/fgvieira/ngsLD) to estimate the decay of linkage disequilibrium (LD) based on genotype likelihoods using sites with 0.01 as minor allele frequency (--min_maf 0.01). We plotted the r2 estimates using the fit_LDdecay.R script provided by ngsLD setting 500 kb as maximum distance between SNPs (--max_kb_dist 500), exhaustive fitting (--fit_level 100) and a fitting bin size of 200 bp (--fit_bin_size 200).

Genome-Wide Association Study

From the genotype likelihoods, we calculated the expected genotypes and used these as allele dosages. Then, we employed the software GEMMA-v0.96 (Zhou & Stephens 2012) to calculate a centered relatedness matrix (-gk 1) excluding SNPs with minor allele frequency below 0.01 (-maf 0.01). Based on ref. (Levi 1996) and the NPA 2010 Book of Standards (Child 2010), we created a phenotype table scoring both the Crest and the FootFeathering traits across the different breeds (Supplementary Spreadsheet). Finally, we used the genetic relatedness matrix to fit a linear mixed model in GEMMA, and performed a likelihood ratio test concerning each of the scored traits separately. Moreover, for both association analysis conducted, we performed a permutation test using MVNpermute (Abney 2015) in order to determine an appropriate significance threshold for each of the association analyses by running 100 permutation replicates and then independently running GEMMA on each permuted phenotype matrix. The p-values from all 100 GEMMA runs were concatenated, and then the 5th percentile p-value, corrected for the total number of tests, was used to draw the lines of significant of each study. Results were plotted using the R package ggman v0.99.0.beta (https://github.com/drveera/ggman).

Supplementary Results & Discussion

Sequencing Output & Data Filtering

We generated 843,216,251 demultiplexed GBS raw reads for 190 samples, representing 61 breeds. The percentage of chimeric reads removed ranged from 0% to 2.65% (average of 0.87%) per sample. The calculated coverage across the regions of interest ranged from 0X to 13.83X (average of 6.12X) among the GBS samples, while it varied from 1.14X to 64.62X (average of 13.20X) among the 52 WGS samples (Supplementary Spreadsheet). Based on a presence/absence heatmap (Supplementary Fig. 2), we excluded 8 samples from downstream analyses (6 GBS and 2 WGS) due to inefficient sequencing. This heatmap shows that there was large discrepancy in the levels of sequencing effectiveness across all predicted cut-sites. An explanation for this could be the large distances between GBS loci that would lead to an indirect size selection at PCR and sequencing steps. In fact, the average distance between every predicted cut-site and the next was 2,785.51 bp. Thus, assuming that fragments longer than 500 bp were excluded during the GBS experimental procedures due to PCR restrictions (extension step lasted 30 sec and the Taq polymerase used is expected to extend 1 kb/min), we expected that roughly 80% of all GBS loci were actually inaccessible as 80.54% of the cut-sites produced fragments greater than 500 bp. This number is very close to our observed missing data value of 74.57% (loci with no data for any sample). Therefore, even though we cannot rule out the role of additional factors behind this observation, we warn that results of in-silico digestion performed as part of a GBS experimental design should be considered with caution as the real sequencing efficiency may be rather different from what was predicted.

Sequence variation between paralogous genomic regions can lead to the identification of false-positive SNPs. Thus, standard genotyping pipelines usually attempt to remove these regions from downstream analyses. The most commonly used method is to remove all regions with extremely high coverage relative to the mean. However, since it is not possible to identify PCR duplicated reads in single-end GBS data, the Global Depth (GD) distribution can be rather misleading. Other methods have been developed to infer paralogous regions in GBS and similar datasets (e.g. (McKinney et al. 2017)) but these methods often assume a natural population in Hardy-Weinberg equilibrium, which is usually not the case for populations of domestic animals due to continuous artificial selection. Thus, since populations of pigeon breeds are indeed not natural populations, we took advantage of the presence of both WGS and GBS samples in our data to develop a new approach to identify paralogs (see Methods), resulting in the exclusion of 0.46% of the loci. In order to validate this approach using the whole dataset, we plotted the average GD for all loci on top of the average GD just for the loci flagged as possible paralogs by our approach (Supplementary Fig. 3b). Intriguingly, the possible paralogous loci identified through the WGS dataset showed no correspondingly high GD on the entire dataset. We believe this is a result of the aforementioned extreme difference of sequencing performance across the cut-sites. Therefore, we conclude that excluding possible paralog loci simply based on higher than expected GD is not efficient and more robust methods should be used whenever feasible. Post-filtering, we retained 354,919 loci (covering roughly 5.87% of the pigeon genome) which were used in all downstream analyses.

Molecular Sexing Based on GBS Data

We used our dataset to evaluate whether GBS-like data would be appropriate to perform molecular sexing as this kind of analysis might be of relevance for future studies. The computed values of the ratio between the coverage of chromosome Z and chromosome 6 calculated for each sample ranged from 0.498 to 1.085 with very few numbers being too far away either from 0.5 or 1.0, thus we could confidently score all samples as either male or female. While 28.2% of the samples scored as female, 71.8% scored as male. Moreover, we noticed some incongruences between our GBS sexing and the phenotype sex noted at the time of sampling, which is consistent with the notorious challenge of sexing most pigeon breeds solely based on morphological and behavioural traits, especially juveniles or young adults. Out of 173 cases for which we had both score types (only samples that passed to the variant calling phase), 14.45% of the cases were incongruent (Supplementary SpreadSheet).

Assessment of the Distribution of GBS Sites Across the Pigeon Genome

To check whether the GBS method produced data at locations spanning the entire pigeon genome, we performed a regression between the size of each scaffold and the number of sites found in each scaffold. There was a strong correlation ($r2 = 0.99$ and p-value $< 2.2e{-16}$) between scaffold size and the number of sites reported (Supplementary Fig. 7a), indicating that the GBS protocol successfully yielded sites randomly distributed across the pigeon genome.

WGS / GBS Samples

Our phylogeny shows that all triplicates cluster together with 100% bootstrap support, confirming the absence of bias when either analysing GBS and WGS data under a single pipeline, or when merging these two data types (Fig. 2). We noted, however, that the GBS libraries systematically presented longer branch lengths with respect to their replicates. This hints at an excess of low frequency SNPs (e.g. singletons and doubletons) in the GBS and WGS-GBS samples. Since these are not shared with the WGS replicate, we conclude they are likely sequencing errors that were considered as alternative alleles (due to the impossibility of removing PCR duplicates). Thus, we believe that the use of a reduced-representation library sequencing method that allows for the removal of PCR duplicates (e.g. paired-end RADseq)

could considerably reduce this methodological issue.

Moreover, we noticed that each WGS-GBS sample invariably had the highest Ho, usually followed by the corresponding GBS library (Supplementary Spreadsheet). Even though we did not investigate this systematic difference in depth, we believe it relates to the previously observed excess of low frequency SNPs on GBS samples. Further investigation will be required to assess the significance of this issue, but we do not believe that it introduced any significant bias in our interpretation of the results. Moreover, although we were unable to identify any other obvious biases concerning the triplicates, we chose to be conservative and kept only the WGS libraries of each triplicate for all downstream analyses (ignoring both GBS and WGS-GBS).

Detailed Phylogenetic Description

The outermost clade of our phylogeny encompasses all Color breeds plus the Frillbacks (Structure), followed by two sister clades with the Jacobin, Old Dutch Capuchine, and Schmalkaldener Mohrenkopf (all Structure), and most Trumpeters. A third clade is formed by the Maltese, King, Polish Lynx and Runt (all Form), plus all Croppers & Pouters, and the Laugher (Trumpeter). The Fantails (Structure), the Shakhsharli (Syrian), the Lahore (Form), the Mookee, and one Iranian Tumbler (both TRHF) form the forth clade, followed by the fifth clade formed by most TRHF, the Cumulet, the Temeschburg Schecken (also known as Timisoara Tumbler), and the Medium-faced Crested Helmet (all part of TRHF). The sixth main clade is formed by the Homers (Form), Carriers, Dragoon (both Wattle), and the Ferals, supporting previous claims that current American feral pigeon populations could have been originated mostly from stray Homers11,12. The Barb, the Spanish Barb and the Scandaroon (all Wattle breeds), together with the Carneau (Form) form a new (i.e. not reported before) sister clade to the sixth clade. The final and seventh clade seen in our phylogeny is formed by the Chinese Owl (Structure), the rest of Owls and the Oriental Frill (both part of Owls & Frills), the Lebanon and Syrian Dewlap (both Syrian), and the Egyptian Swift (Form).

Within/Between Genetic Distances of Pigeon Breeds

In order to better identify putative phylogenetic outliers of each breed, we summarized all the pairwise genetic distances on a violin plot (Supplementary Fig. 4), clustering the data into 4 categories: Intra-replicates (distances among WGS, GBS and WGS-GBS triplicates), Intra-breeds (distances among individuals within each breed), Inter-breeds (distances among individuals belonging to different breeds) and Inter-species (distances from all pigeon samples to the outgroup). As seen in the phylogeny, samples IndianFantail_03 and IranianTumbler_01 fall within the Cumulet and the Shakhsharli clades, respectively. The pattern can be seen even more clearly in the violin plot (Supplementary Fig. 4), where these samples show very high intra-breed distances (at an inter-breed level), and low inter-breed distances to the Cumulet and Shakhsharli, respectively (at an intra-breed level). Interestingly, despite the fact that IndianFantail_03 sample was deemed an Indian Fantail by its owner, the breeder also reported that this pigeon had substantial Cumulet ancestry. On the other hand, given their shared geographic region of origin, the close genetic similarity between the IranianTumbler_01 and the Shakhsharli breed is completely expected.

Another unexpected result is the very low intra-breed genetic distances (at a replicate level) between Fantails 02 and 09, and between Scandaroons 01 and 02. These results could indicate that these samples either derive from the same individuals, identical twins, or siblings of highly related parents (breeders often breed for several years from a pair that produces good offspring). Since these four pigeons had different leg-band numbers (a unique tag for each bird that is not changeable or removable) and avian identical twins are extremely rare70, we postulate that these samples might in fact represent siblings from closely related parents (e.g. the Fantails belonged to the same breeder). Finally, there are three pairs of breeds that show very high genetic similarity (at an intra-breed level): Jacobins

and Old Dutch Capuchines, English Long-faced Tumbler and Parlor Roller, and Laugher and Marchenero Pouter. The two breeds in the first pair are believed to share a common origin and are also morphologically similar (e.g. both present a well-developed hood), thus it is not t surprising that they are genetically so similar. The second pair, both belonging to the TRHF group, thus we speculate this could be the result of shared ancestry or recent inter-breed cross. In fact, it has been reported that dog breeds outside their country of origin i) experience a loss in genetic diversity due to importation bottleneck, and ii) experience more inter-breed admixture, since breeders in the importing countries tend to be more prone to perform experimental crosses15. This could well be similar with pigeon breeds. Thus, since most of our samples were collected in the USA, which is not the native country for the majority of our breeds, the pigeons within our panel of samples might be more outbred than pigeons belonging to the same breeds in their respective country of origin.

Abnormal Samples

According to the phylogeny, the sample IndianFantail_03 does not share proportions of individual ancestries with nor is located near the remaining group of Indian Fantails on the MDS. Instead, this sample shares considerable genetic similarity with the Cumulet group, corroborating what was previously seen on the phylogeny (Fig. 2) and violin plot (Supplementary Fig. 4). In the same way, the two Iranian Tumbler samples do not show similar proportions of individual ancestries and are also located on different regions of MDS. Unfortunately, no photo voucher exists for the IranianTumbler_02 sample, thus we cannot fully exclude the possibility of mislabelling issues. However, we highlight that this sample was collected at a breeder's house, therefore it is plausible that it was an experimental, hence out-crossed bird.

Notes on the NPA Classification

Based on the results of the phylogenetic, Admixture and MDS analyses, we are in a position to highlight some points regarding the current NPA classification: the Chinese Owl shows higher genomic proximity to the Owls & Frills group than to the Structure, while the Egyptian Swift to the Syrian group than to the Form. As for those breeds that are not currently recognized by the NPA, our results demonstrate that the Birmingham Roller and the Backa Tumbler share the highest genomic similarity with the TRHF group, the California Color Pigeon and the Saxon Fairy Swallow with the Color, and the Mindian Fantail with the Structure.

Linkage Disequilibrium Levels Across Pigeon Breeds

Patterns of Linkage Disequilibrium (LD) decay can greatly affect the efficiency of Genome-Wide Association Studies (GWAS). While this has been previously explored using a reduced number of samples and breeds (Shapiro et al. 2013), we took advantage of our expanded dataset to improve estimates of pigeon LD decay, something that may help clarify the appropriateness of different pigeon datasets in studies attempting to link genetic variation with phenotypic variability. Specifically we used all samples in Dataset 3 (only excluding the outgroup) to calculate r2, but focussed on the 0.95 quantile since these data points are most relevant for GWAS. Our analyses (Supplementary Fig. 10) yield higher LD estimates among pigeon breeds than those previously reported (Shapiro et al. 2013), with median relevant LD (Aerts et al. 2007) decaying at around 30 kb, but possibly extending until 200 kb due to high variance.

Although the average inter-SNP distance is approximately 17 kb (Supplementary Fig. 7b), we conclude that the levels of long range LD found here indicate that our marker density is appropriate for GWAS based on GBS data.

Assessing GBS Performance for Genome-wide Association Studies

Despite the sparse set of markers that are intrinsically generated by reduced-library sequencing protocols, several studies have demonstrated that this kind of data can be used to successfully perform GWAS (e.g. ref. (Parker et al. 2016; Otto et al. 2017; Barría et al. 2018)

). In this way, we wished to investigate whether GWAS analyses on our GBS-derived panel of SNPs would be able to recapitulate the results of previous analyses undertaken on WGS data. Thus, we undertook a GWAS analysis using Dataset 3 targeting two morphological traits for which the genomic basis was already reconstructed using WGS. A previous study (Domyan et al. 2016) described two haplotypes (that include regions: Scaffold_79:6719000-6763000 in Cliv_1.0, ScoHet5_1033.2:7486712-7530712 in Cliv_2.1; and Scaffold_70:731300 in Cliv_1.0, ScoHet5_149:1203848 in Cliv_2.1) to be linked to the FootFeathering trait. In our dataset we found three SNPs on scaffold ScoHet5_1033.2 and one SNP on scaffold ScoHet5_149 to be significantly associated with this trait (Supplementary Fig. 11a). Remarkably, the three SNPs on scaffold ScoHet5_1033.2 were at approximately 135 kb from the known haplotype, while the single SNP on scaffold ScoHet5_149 was at approximately 300 kb from the other known haplotype. Thus, considering the intrinsic scale of each method, our GBS-based GWAS was able to identify the same genomic regions involved in the FootFeathering trait that were previously reported. Intriguingly, we also found a SNP (ScoHet5_205:16892632) on a third scaffold significantly associated with this trait at a marginal level. We speculate that it represents either i) a false positive, ii) a scaffold physically close to one of the two known haplotypes associated with this trait, iii) a third but yet unidentified locus that contributes to the trait. As for the Crest trait, an 11-kb haplotype containing a putatively causative SNV (Scaffold_612:596613 in Cliv_1.0; ScoHet5_3280:262233 in Cliv_2.1) is thought to be associated with this trait (Shapiro et al. 2013). Intriguingly, no SNP in our dataset was significantly associated with this trait (Supplementary Fig. 11b). However, there is a markedly low density of GBS cut-sites (and hence SNPs) around the region in question (only 3 SNPs less than 300 kb away), leading to a considerably greater SNP spacing in relation to the haplotype we are trying to detect.

Our results corroborate previous studies which demonstrate that it is somewhat possible to perform GWAS based on reduced-representation library data (e.g. (Parker et al. 2016; Otto et al. 2017; Barría et al. 2018)). Nonetheless, given the wide SNP spacing produced by this sort of method (e.g. GBS), it is important to highlight that the power of this type of analysis will be strongly dependent on the average distance between SNPs, as well as levels of LD. In this regard, protocols with lower levels of missing-data (e.g. paired-end and double-digestion RADseq) are expected to perform considerably better.

Supplementary References

Abney M. 2015. Permutation testing in the presence of polygenic variation. Genet. Epidemiol. 39:249–258.

Aerts J et al. 2007. Extent of linkage disequilibrium in chicken. Cytogenet. Genome Res. 117:338–345.

Barría A et al. 2018. Genomic Predictions and Genome-Wide Association Study of Resistance Against Piscirickettsia salmonis in Coho Salmon (Oncorhynchus kisutch) Using ddRAD Sequencing. G3 . 8:1183–1194.

Camacho C et al. 2009. BLAST+: architecture and applications. BMC Bioinformatics. 10:421.

Damas J et al. 2017. Upgrading short-read animal genome assemblies to chromosome level using comparative genomics and a universal probe set. Genome Res. 27:875–884.

Domyan ET et al. 2016. Molecular shifts in limb identity underlie development of feathered feet in two domestic avian species. Elife. 5:e12115.

Levi WM. 1996. Encyclopedia of Pigeon Breeds. TFH Publications.

McKinney GJ, Waples RK, Seeb LW, Seeb JE. 2017. Paralogs are revealed by proportion of heterozygotes and deviations in read ratios in genotyping-by-sequencing data from natural populations. Mol. Ecol. Resour. 17:656–669.

National Pigeon Association. 2010. 2010 National Pigeon Association Book of Standards. Purebred Pigeon Publishing.

Otto L-G et al. 2017. Use of genotyping-by-sequencing to determine the genetic structure in the medicinal plant chamomile, and to identify flowering time and alpha-bisabolol associated SNP-loci by genome-wide association mapping. BMC Genomics. 18:599.

Parker CC et al. 2016. Genome-wide association study of behavioral, physiological and gene expression traits in outbred CFW mice. Nat. Genet. 48:919–926.

Shapiro MD et al. 2013. Genomic diversity and evolution of the head crest in the rock pigeon. Science. 339:1063–1067.

Zhou X, Stephens M. 2012. Genome-wide efficient mixed-model analysis for association studies. Nat. Genet. 44:821–824.

Supplementary Figures

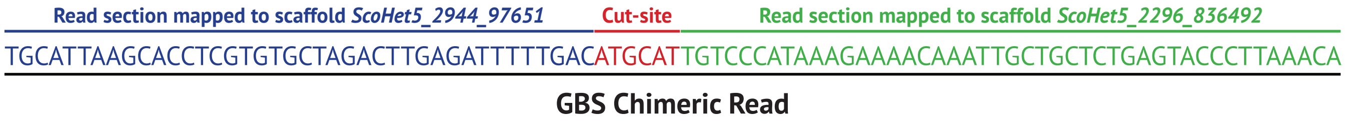

Supplementary Fig. 1. Schematic example of a GBS chimeric read.

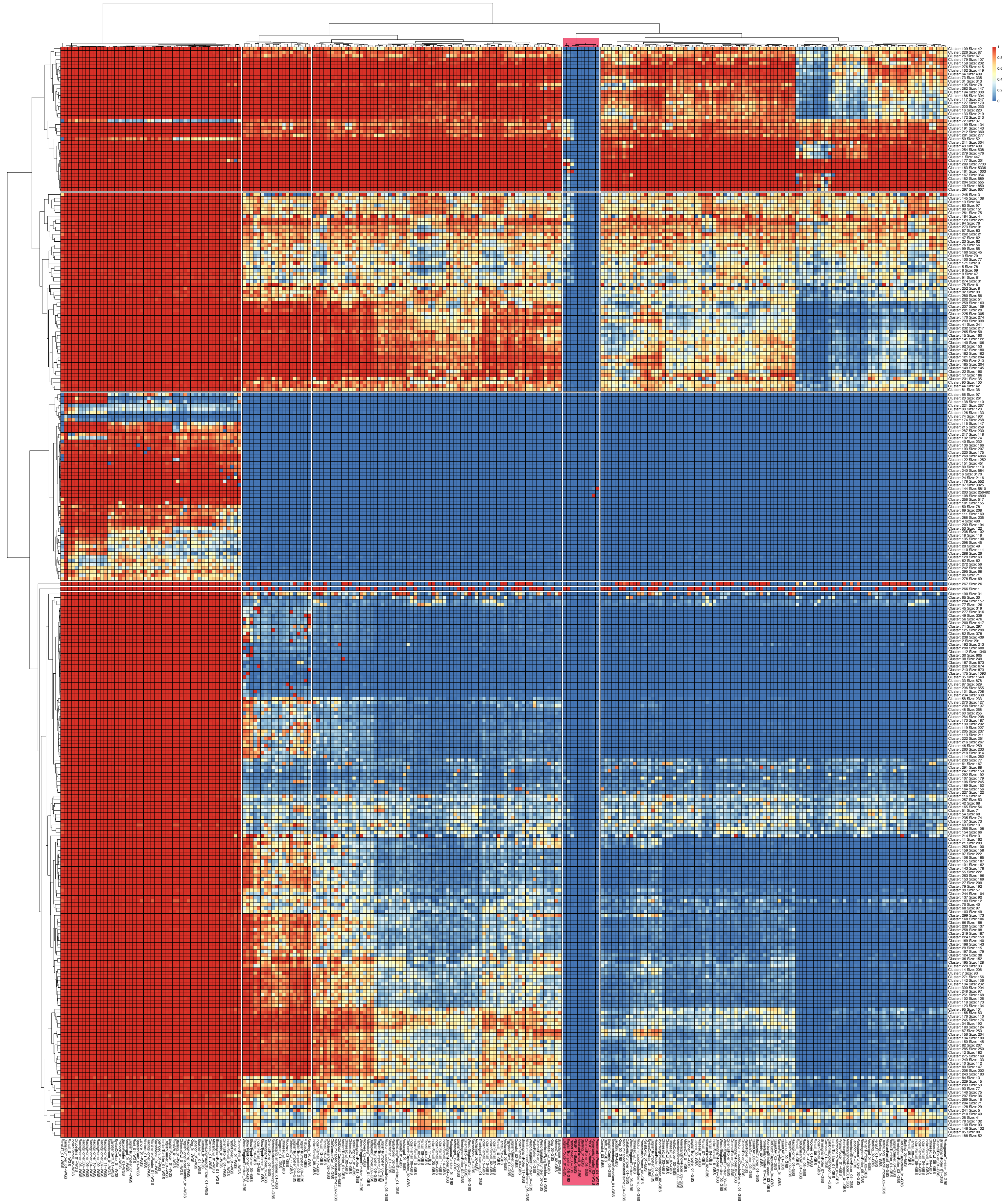

Supplementary Fig. 2. Heatmap based on the presence/absence matrix. Columns represent individual samples, while rows represent clusters of loci. Those samples that failed to produce sufficient GBS reads and blanks samples are marked in red.

A)

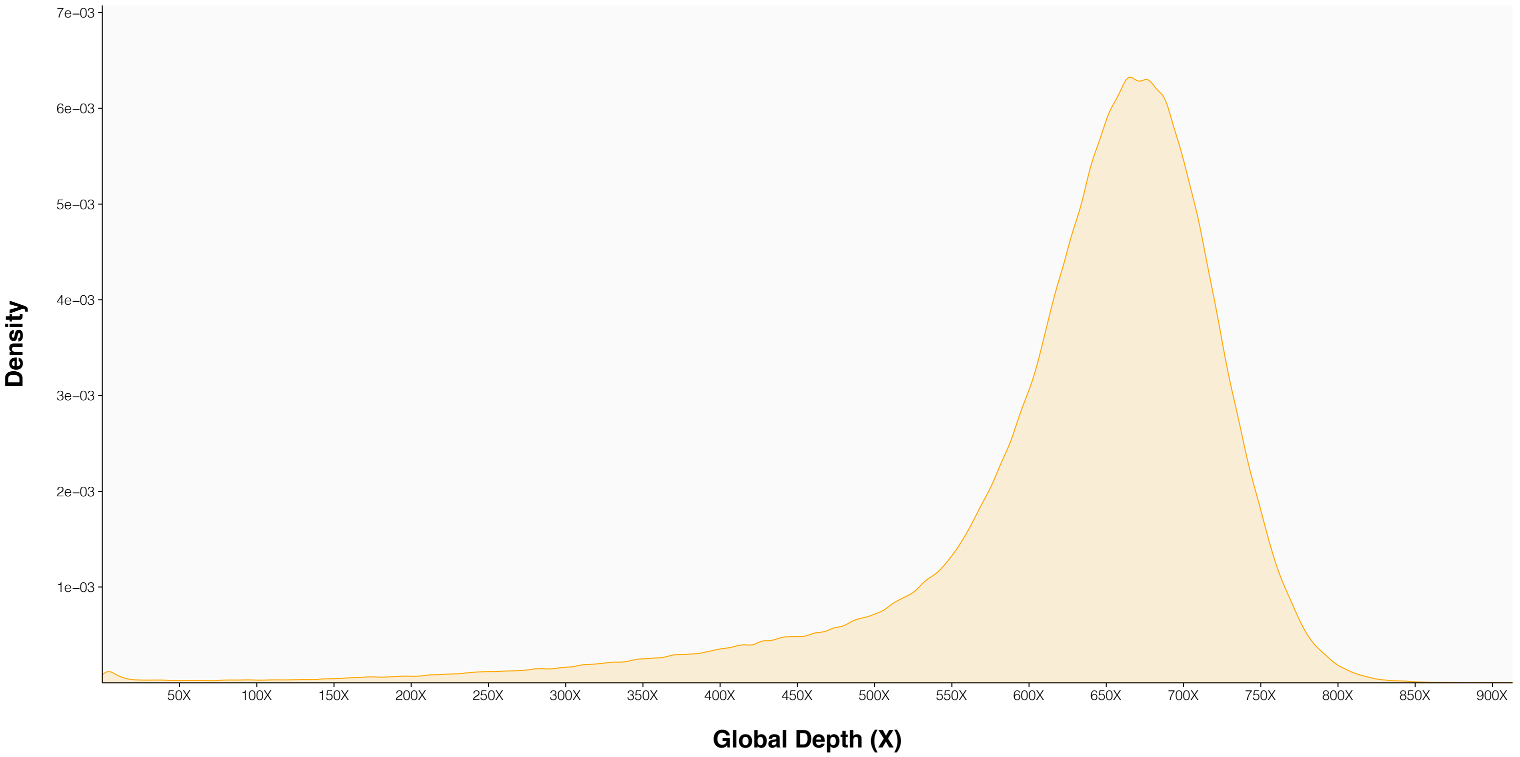

B)

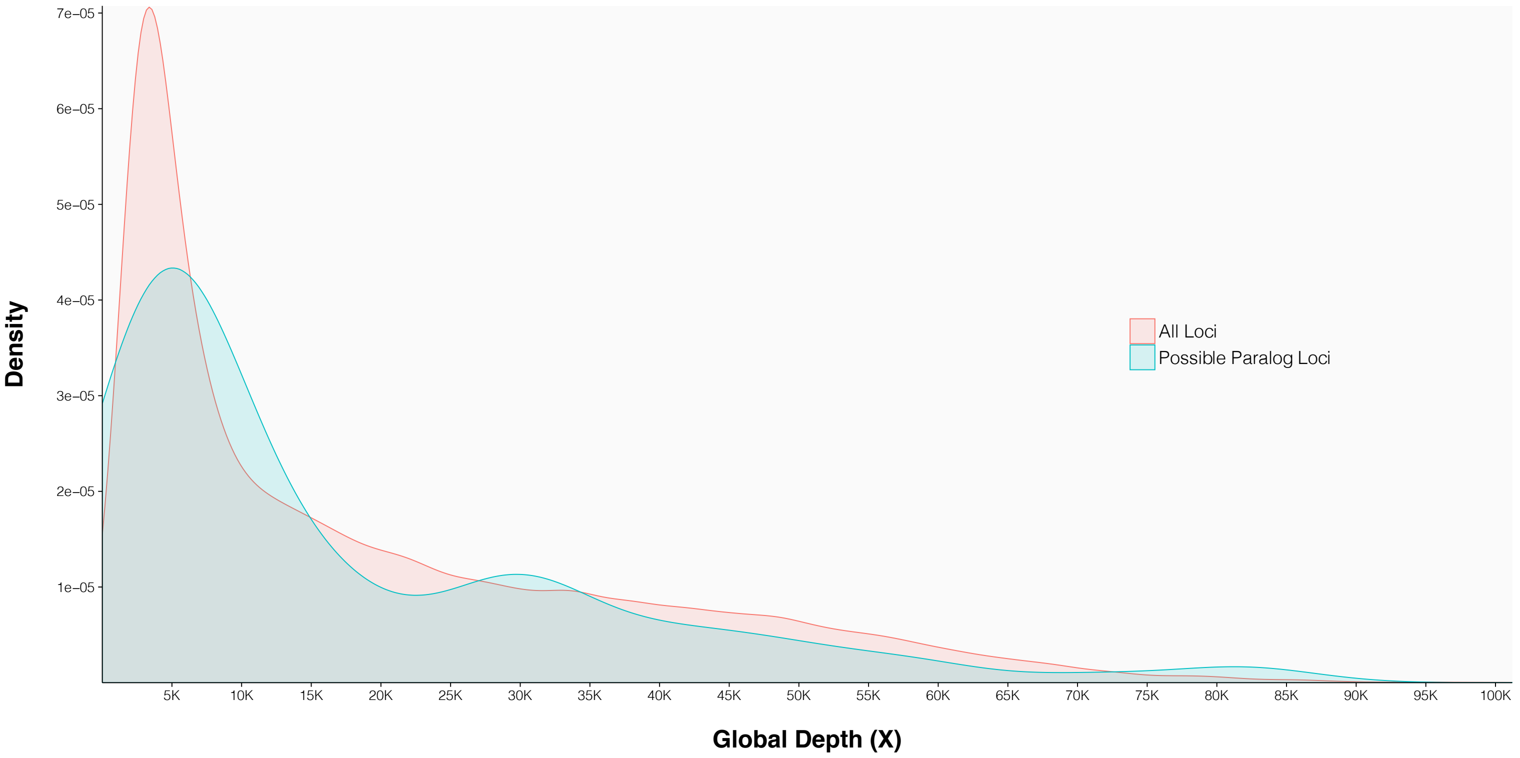

Supplementary Fig. 3. Global Depth (GD) density plots. (*a*) for all the 50 WGS samples that passed our initial filter. (*b*) for all samples that passed our initial filter (in red). A subset of this data only containing the possible paralog loci is also plotted (in light blue).

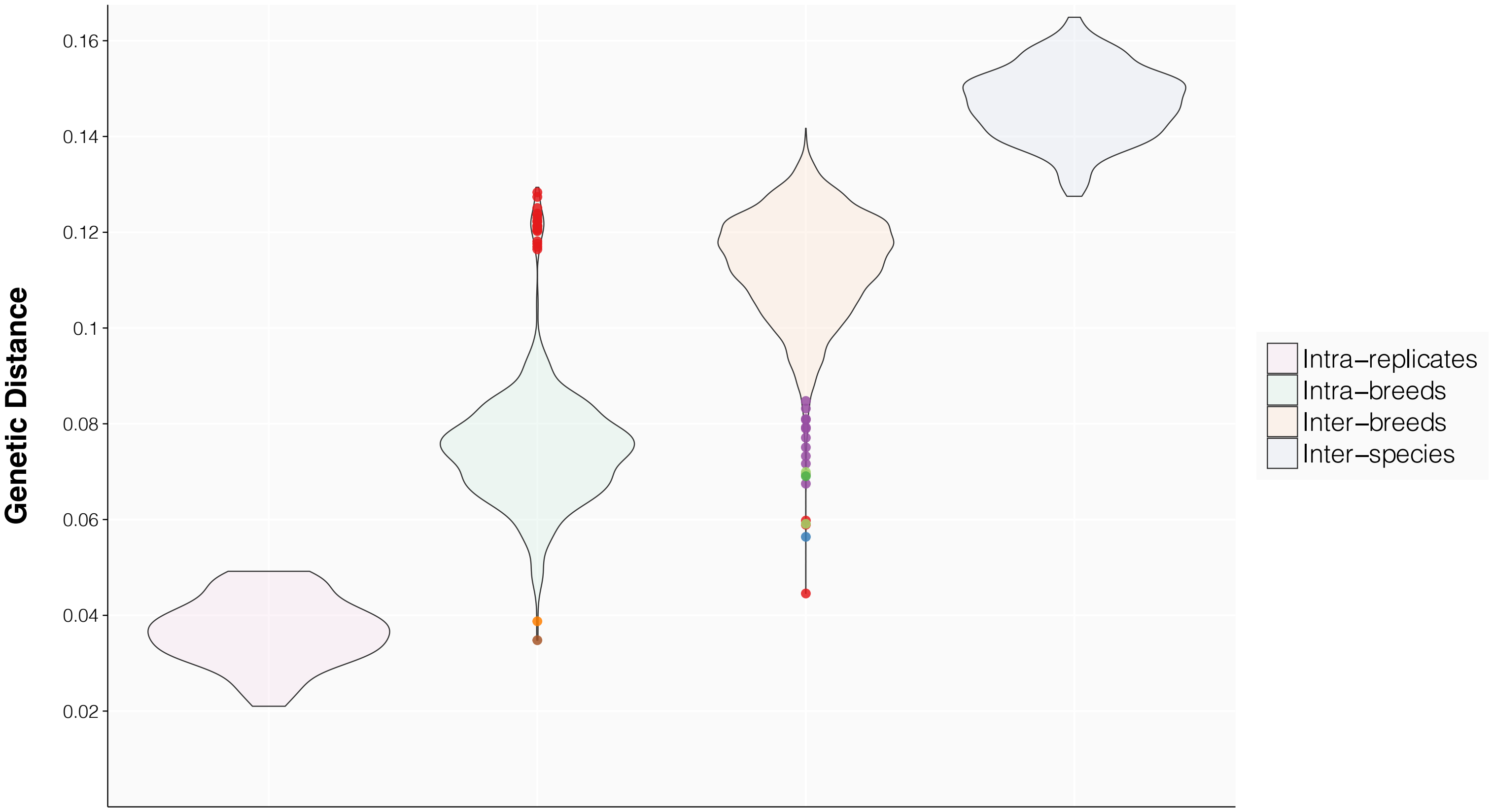

Supplementary Fig. 4. Violin plot of genetic distances. Coloured dots represent pairwise comparisons between IndianFantail_03 and all other Indian Fantails (red dots; Intra-breeds), IndianFantail_03 and all Cumulets (red dots; Inter-breeds), Laugher_01 and Shakhsharli_01 (blue dot), Fantail_02 and Fantail_09 (orange dot), Scandaroon_01 and Scandaroon_02 (brown dot), all Jacobins and all Old Dutch Capuchines (purple dots), EnglishLongFacedTumbler_01 and all Parlor Rollers (light green dots) and Laugher_01 and MarcheneroPouter_01 (dark green dot). The width of each plot is proportional to the number of samples at a given genetic distance.

A)

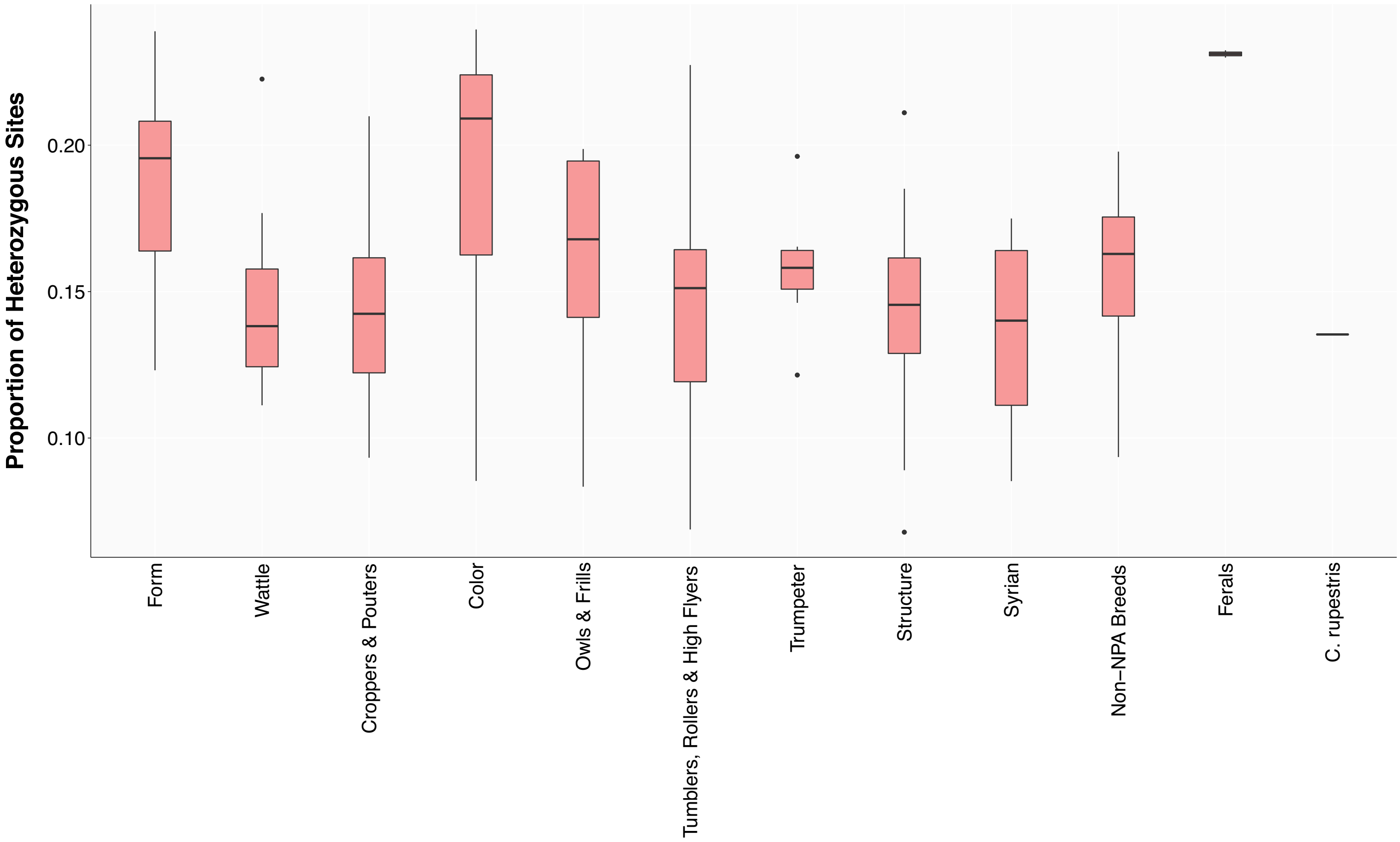

B)

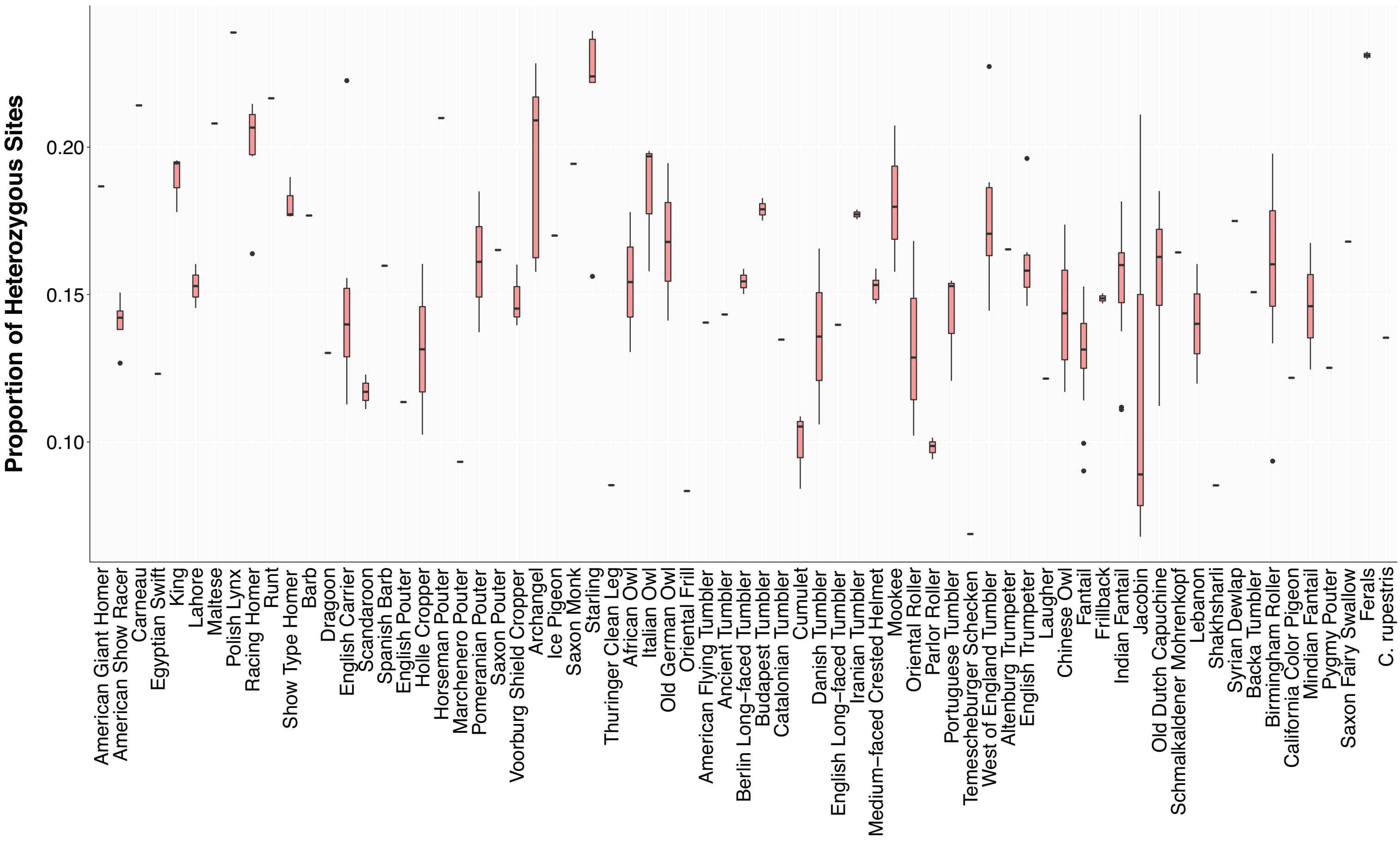

Supplementary Fig. 5. Proportion of observed heterozygous sites. (*A*) plotted per NPA group. (*B*) plotted per breed. All absolute values can be found in the Supplementary Spreadsheet.

A)

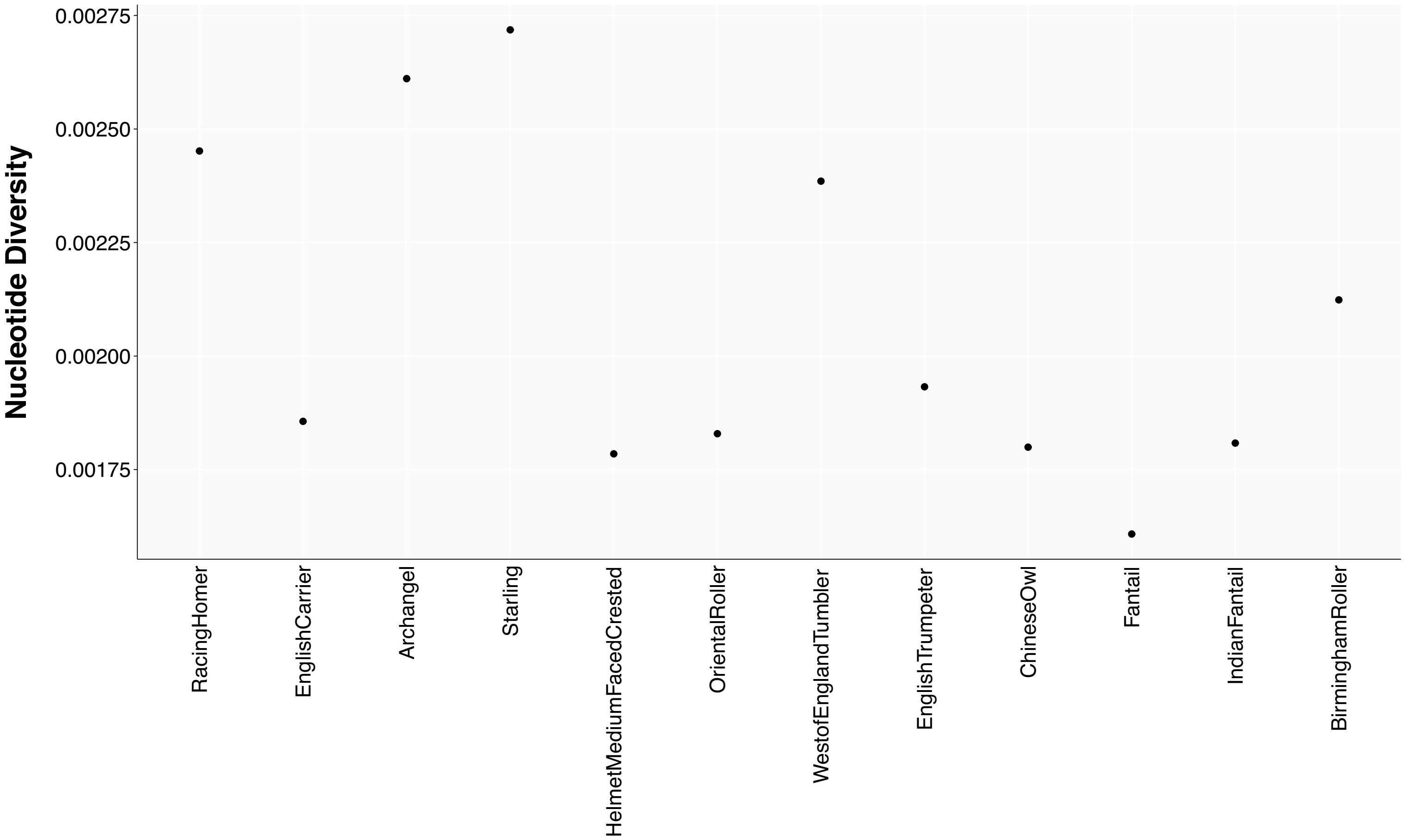
Nucleotide Diversity
0.00275
0.00250
0.00225
0.00200
0.00175
RacingHomer
EnglishCarrier
Archangel
Starling
HelmetMediumFacedCrested
OrientalRoller
WestofEnglandTumbler
EnglishTrumpeter
ChineseOwl
Fantail
IndianFantail
BirminghamRoller

B)

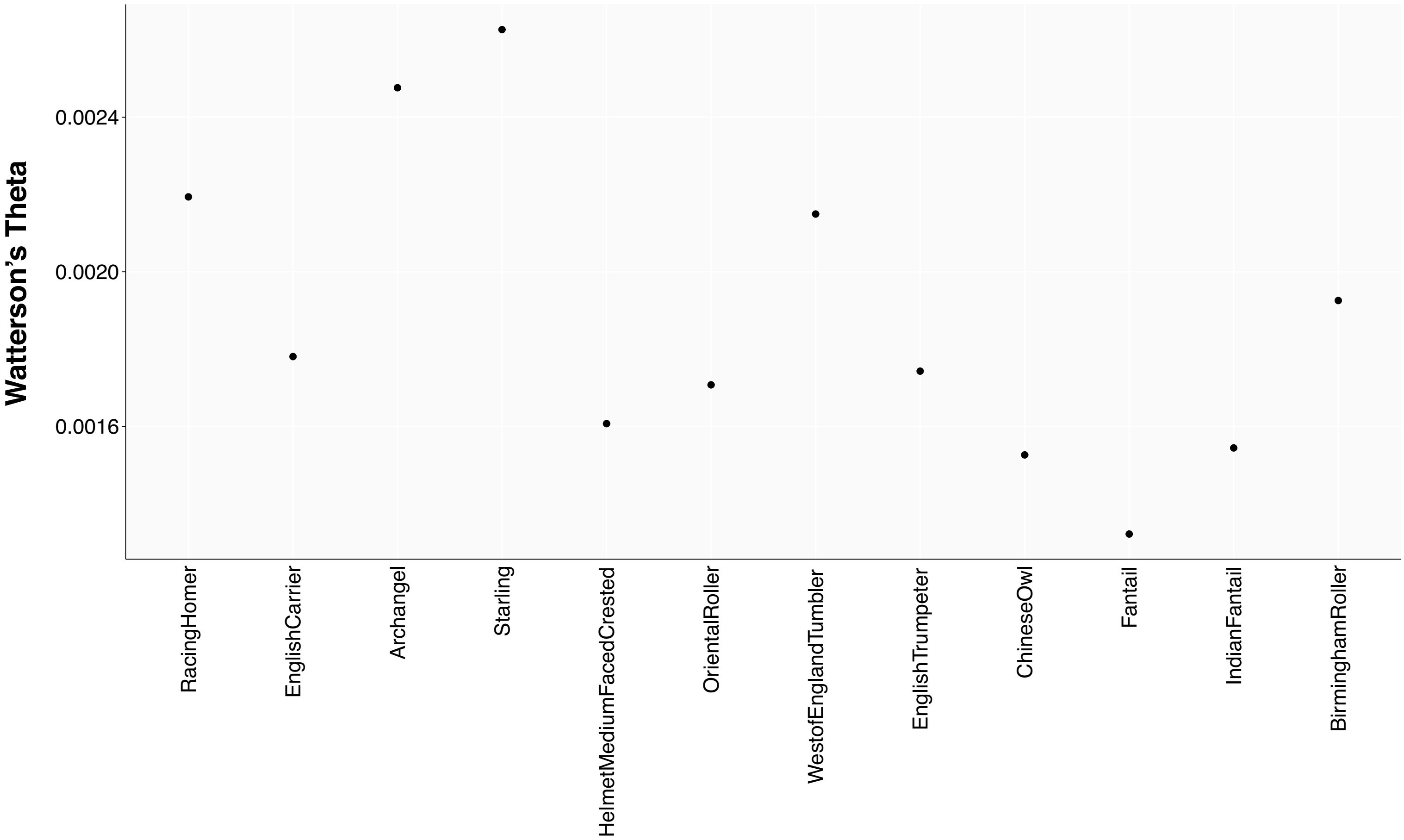
Watterson's Theta
0.0024
0.0020
0.0016
RacingHomer
EnglishCarrier
Archangel
Starling
HelmetMediumFacedCrested
OrientalRoller
WestofEnglandTumbler
EnglishTrumpeter
ChineseOwl
Fantail
IndianFantail
BirminghamRoller

C)

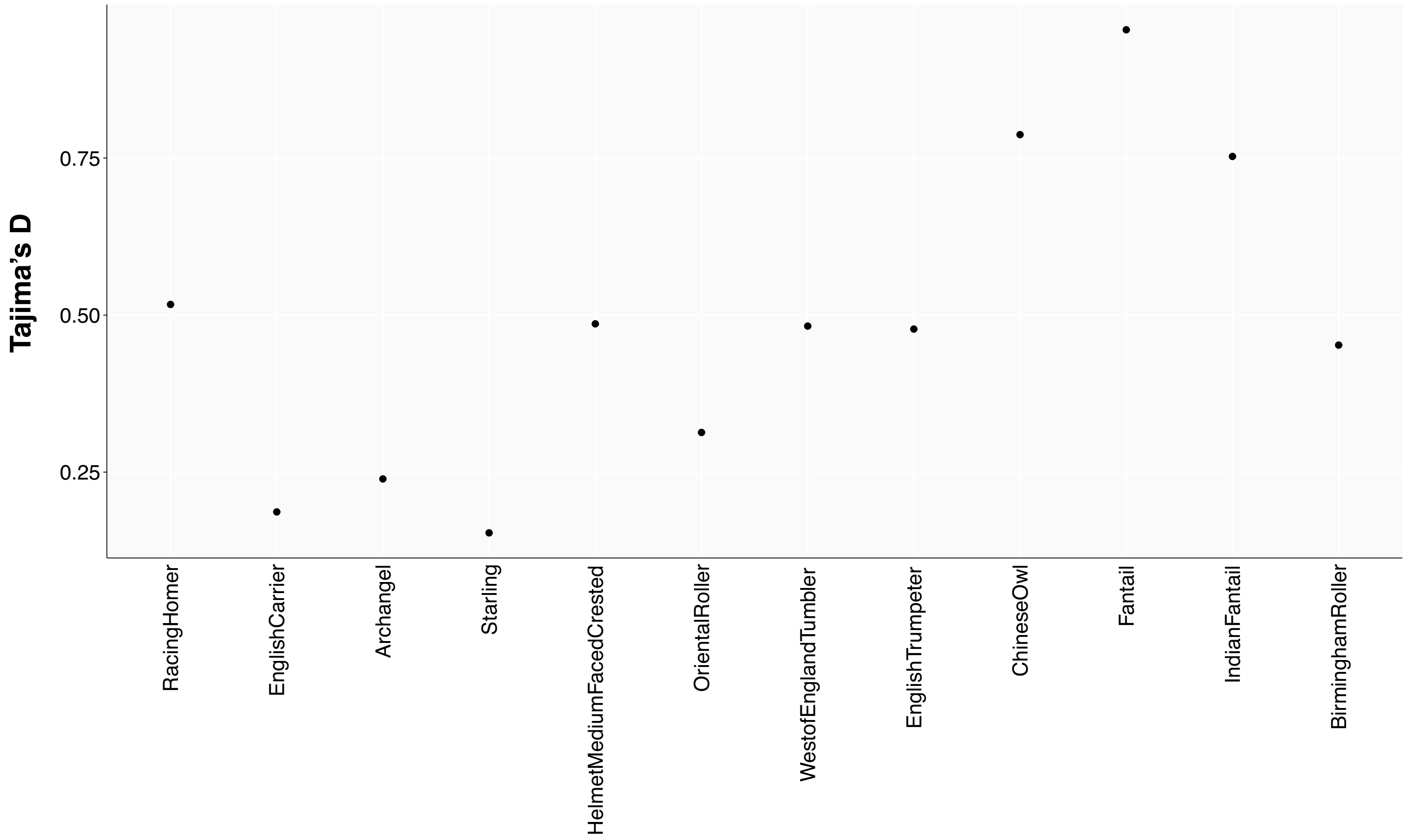

Supplementary Fig. 6. Population genetics estimates per breed. (*A*) Nucleotide Diversity (π)**.** (*B*) Watterson's Theta (θw). (*C*) Tajima's D. All absolute values can be found in the Supplementary Spreadsheet.

A)

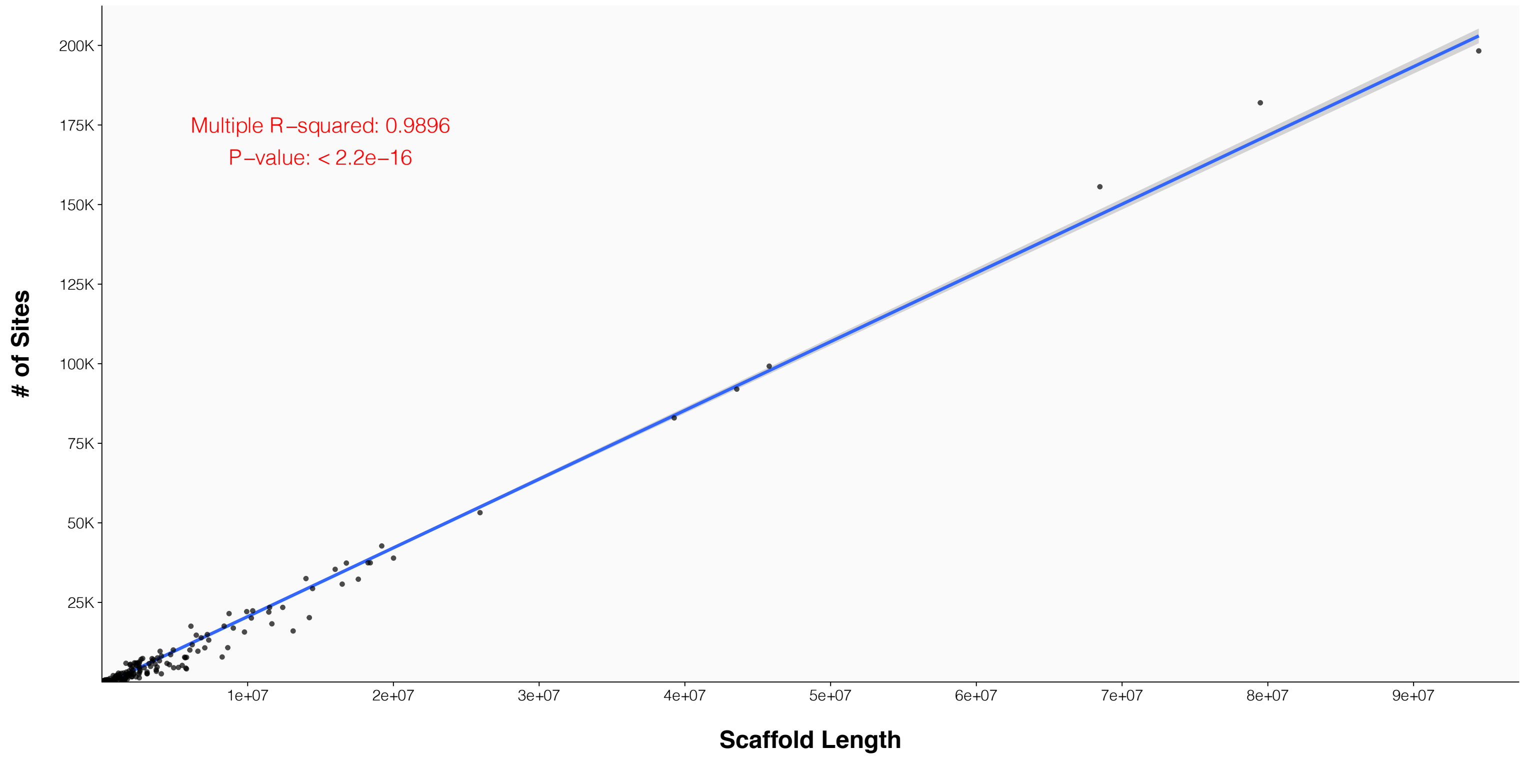

B)

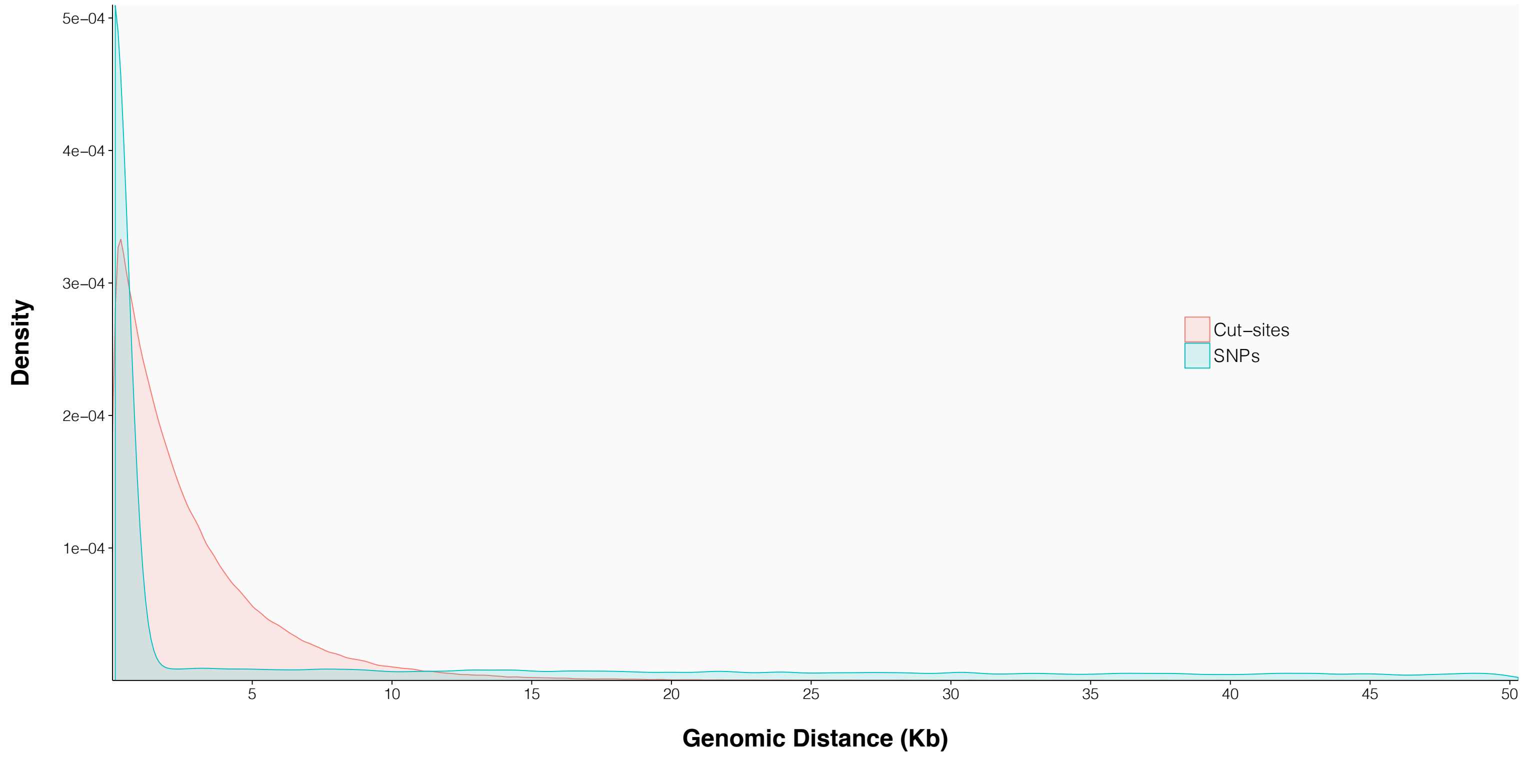

Supplementary Fig. 7. Sites and SNPs information. (*A*) Regression analysis plot showing the correlation between the scaffold sizes and number of sites found in each scaffold. (*B*) Density plot comparing the distances among cut-sites (in red) and among SNPs (in light blue).

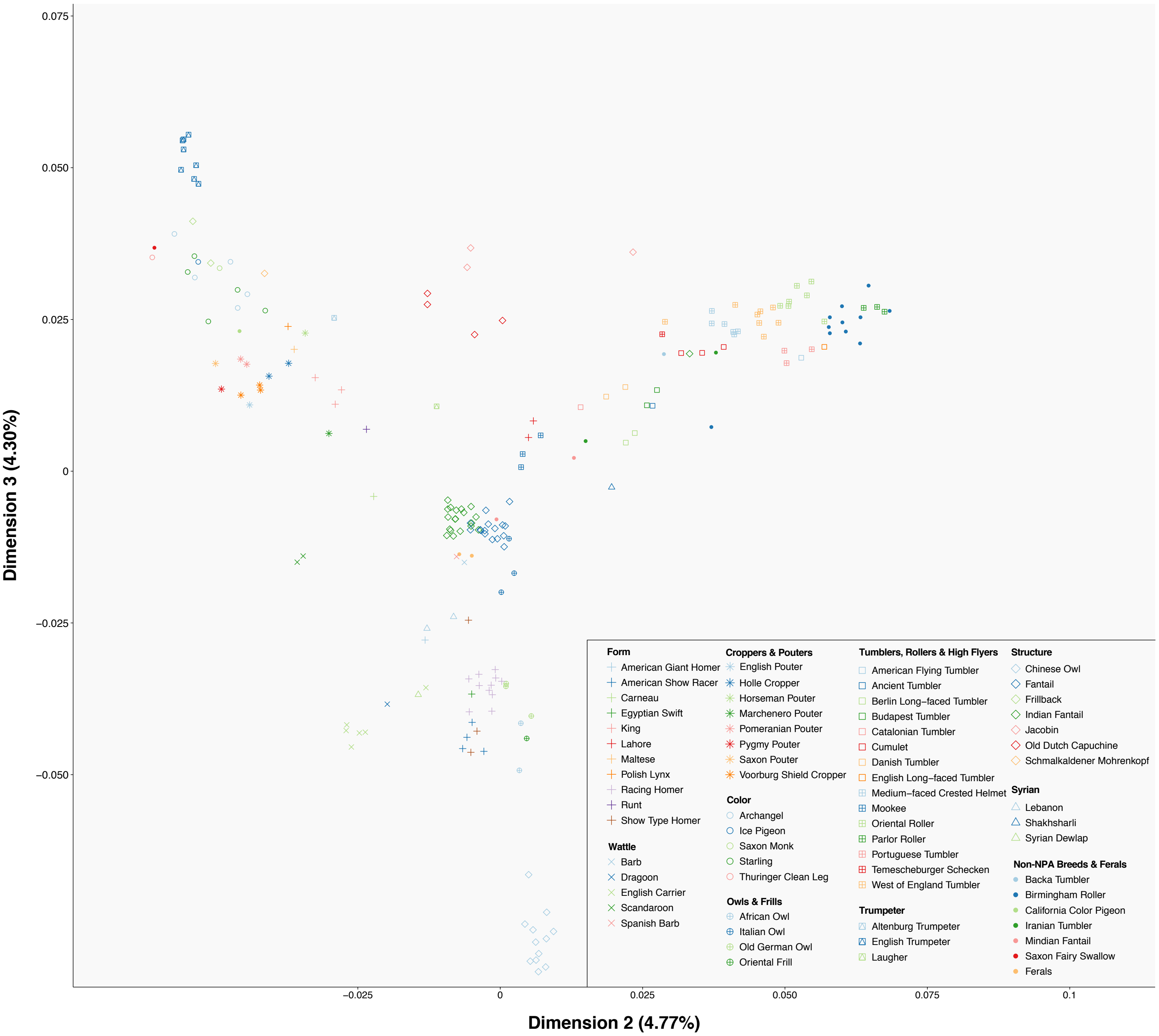

Supplementary Fig. 8. Multidimensional Scaling Analysis (MDS). Dimensions 2 and 3 of are plotted and each point on the plot represent a single individual.

A)

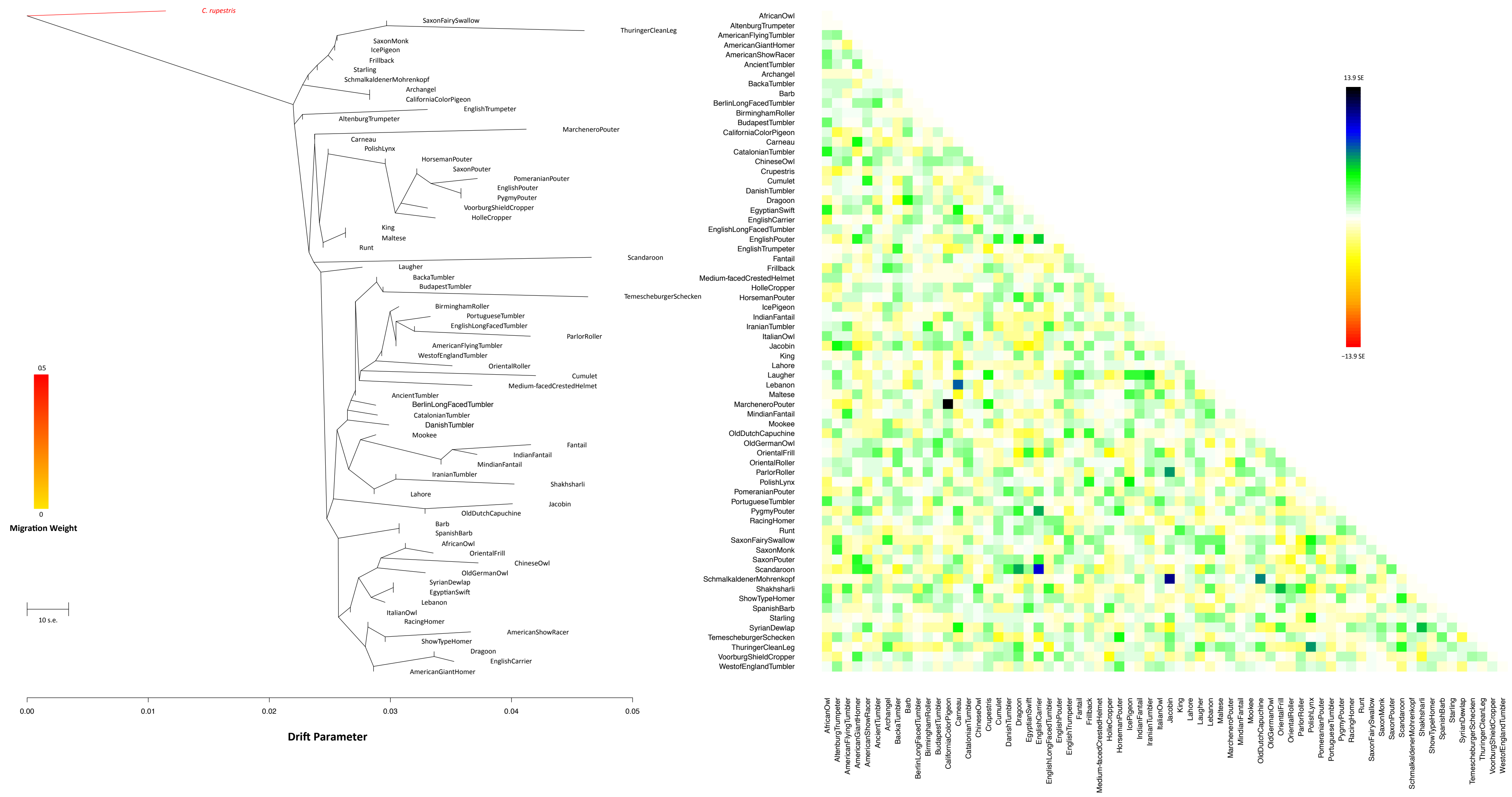
C. rupestris
SaxonFairySwallow
ThuringerCleanLeg
SaxonMonk
IcePigeon
Frillback
Starling
SchmalkaldenerMohrenkopf
Archangel
CaliforniaColorPigeon
EnglishTrumpeter
AltenburgTrumpeter
MarcheneroPouter
Carneau
PolishLynx
HorsemanPouter
SaxonPouter
PomeranianPouter
EnglishPouter
PygmyPouter
VoorburgShieldCropper
HolleCropper
King
Maltese
Runt
Scandaroon
Laugher
BackaTumbler
BudapestTumbler
TemescheburgerSchecken
BirminghamRoller
PortugueseTumbler
EnglishLongFacedTumbler
ParlorRoller
AmericanFlyingTumbler
WestofEnglandTumbler
OrientalRoller
Cumulet
Medium-facedCrestedHelmet
AncientTumbler
BerlinLongFacedTumbler
CatalonianTumbler
DanishTumbler
Mookee
Fantail
IndianFantail
MindianFantail
IranianTumbler
Shakhsharli
Lahore
Jacobin
OldDutchCapuchine
Barb
SpanishBarb
AfricanOwl
OrientalFrill
ChineseOwl
OldGermanOwl
SyrianDewlap
EgyptianSwift
Lebanon
ItalianOwl
RacingHomer
AmericanShowRacer
ShowTypeHomer
Dragoon
EnglishCarrier
AmericanGiantHomer
0.5
0
Migration Weight
10 s.e.
0.00
0.01
0.02
0.03
0.04
0.05
Drift Parameter
13.9 SE
−13.9 SE

B)

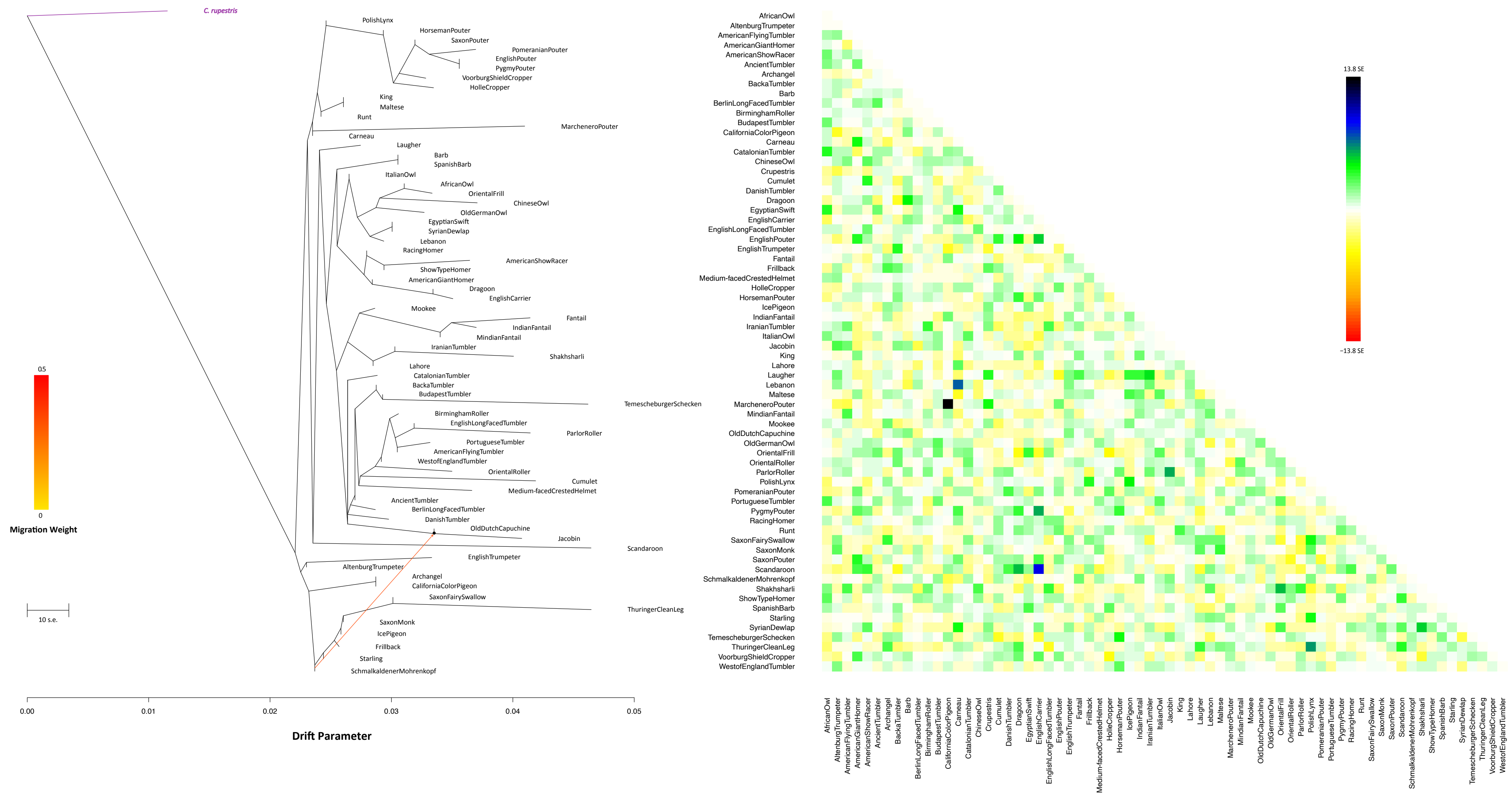
C. rupestris
PolishLynx
HorsemanPouter
SaxonPouter
PomeranianPouter
EnglishPouter
PygmyPouter
VoorburgShieldCropper
HolleCropper
King
Maltese
Runt
MarcheneroPouter
Carneau
Laugher
Barb
SpanishBarb
ItalianOwl
AfricanOwl
OrientalFrill
ChineseOwl
OldGermanOwl
EgyptianSwift
SyrianDewlap
Lebanon
RacingHomer
AmericanShowRacer
ShowTypeHomer
AmericanGiantHomer
Dragoon
EnglishCarrier
Mookee
Fantail
IndianFantail
MindianFantail
IranianTumbler
Shakhsharli
Lahore
CatalonianTumbler
BackaTumbler
BudapestTumbler
TemescheburgerSchecken
BirminghamRoller
EnglishLongFacedTumbler
ParlorRoller
PortugueseTumbler
AmericanFlyingTumbler
WestofEnglandTumbler
OrientalRoller
Cumulet
Medium-facedCrestedHelmet
AncientTumbler
BerlinLongFacedTumbler
DanishTumbler
OldDutchCapuchine
Jacobin
Scandaroon
EnglishTrumpeter
AltenburgTrumpeter
Archangel
CaliforniaColorPigeon
SaxonFairySwallow
ThuringerCleanLeg
SaxonMonk
IcePigeon
Frillback
Starling
SchmalkaldenerMohrenkopf
0.5
0
Migration Weight
10 s.e.
0.00
0.01
0.02
0.03
0.04
0.05
Drift Parameter
13.8 SE
−13.8 SE
AfricanOwl
AltenburgTrumpeter
AmericanFlyingTumbler
AmericanGiantHomer
AmericanShowRacer
AncientTumbler
Archangel
BackaTumbler
Barb
BerlinLongFacedTumbler
BirminghamRoller
BudapestTumbler
CaliforniaColorPigeon
Carneau
CatalonianTumbler
ChineseOwl
Crupestris
Cumulet
DanishTumbler
Dragoon
EgyptianSwift
EnglishCarrier
EnglishLongFacedTumbler
EnglishPouter
EnglishTrumpeter
Fantail
Frillback
Medium-facedCrestedHelmet
HolleCropper
HorsemanPouter
IcePigeon
IndianFantail
IranianTumbler
ItalianOwl
Jacobin
King
Lahore
Laugher
Lebanon
Maltese
MarcheneroPouter
MindianFantail
Mookee
OldDutchCapuchine
OldGermanOwl
OrientalFrill
OrientalRoller
ParlorRoller
PolishLynx
PomeranianPouter
PortugueseTumbler
PygmyPouter
RacingHomer
Runt
SaxonFairySwallow
SaxonMonk
SaxonPouter
Scandaroon
SchmalkaldenerMohrenkopf
Shakhsharli
ShowTypeHomer
SpanishBarb
Starling
SyrianDewlap
TemescheburgerSchecken
ThuringerCleanLeg
VoorburgShieldCropper
WestofEnglandTumbler

C)

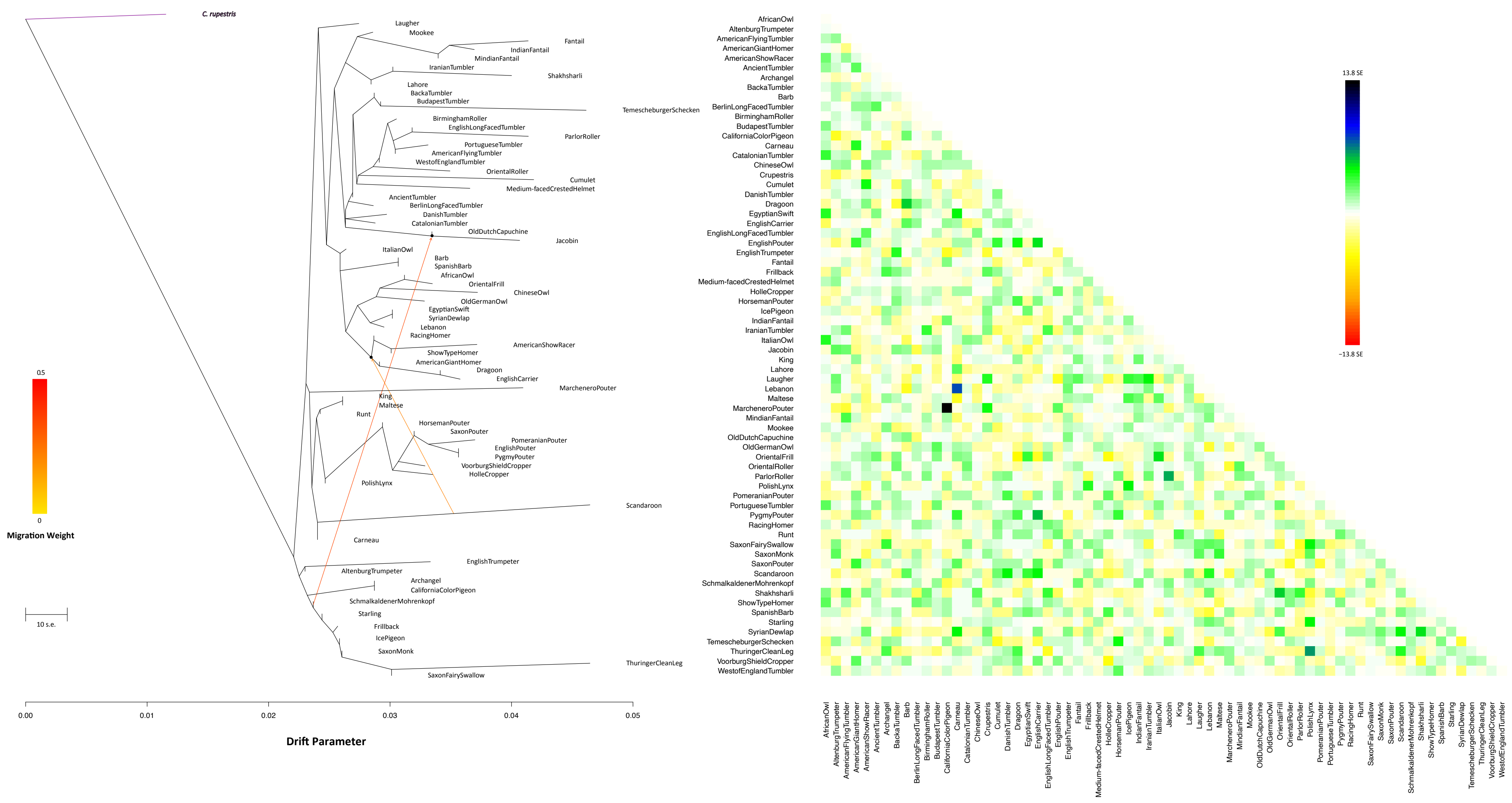
C. rupestris
Laugher
Mookee
Fantail
IndianFantail
MindianFantail
IranianTumbler
Shakhsharli
Lahore
BackaTumbler
BudapestTumbler
TemescheburgerSchecken
BirminghamRoller
EnglishLongFacedTumbler
ParlorRoller
PortugueseTumbler
AmericanFlyingTumbler
WestofEnglandTumbler
OrientalRoller
Cumulet
Medium-facedCrestedHelmet
AncientTumbler
BerlinLongFacedTumbler
DanishTumbler
CatalonianTumbler
OldDutchCapuchine
Jacobin
ItalianOwl
Barb
SpanishBarb
AfricanOwl
OrientalFrill
ChineseOwl
OldGermanOwl
EgyptianSwift
SyrianDewlap
Lebanon
RacingHomer
AmericanShowRacer
ShowTypeHomer
AmericanGiantHomer
Dragoon
EnglishCarrier
MarcheneroPouter
King
Maltese
Runt
HorsemanPouter
SaxonPouter
PomeranianPouter
EnglishPouter
PygmyPouter
VoorburgShieldCropper
HolleCropper
PolishLynx
Scandaroon
Carneau
EnglishTrumpeter
AltenburgTrumpeter
Archangel
CaliforniaColorPigeon
SchmalkaldenerMohrenkopf
Starling
Frillback
IcePigeon
SaxonMonk
ThuringerCleanLeg
SaxonFairySwallow
0.5
0
Migration Weight
10 s.e.
0.00
0.01
0.02
0.03
0.04
0.05
Drift Parameter
13.8 SE
−13.8 SE
AfricanOwl
AltenburgTrumpeter
AmericanFlyingTumbler
AmericanGiantHomer
AmericanShowRacer
AncientTumbler
Archangel
BackaTumbler
Barb
BerlinLongFacedTumbler
BirminghamRoller
BudapestTumbler
CaliforniaColorPigeon
Carneau
CatalonianTumbler
ChineseOwl
Crupestris
Cumulet
DanishTumbler
Dragoon
EgyptianSwift
EnglishCarrier
EnglishLongFacedTumbler
EnglishPouter
EnglishTrumpeter
Fantail
Frillback
Medium-facedCrestedHelmet
HolleCropper
HorsemanPouter
IcePigeon
IndianFantail
IranianTumbler
ItalianOwl
Jacobin
King
Lahore
Laugher
Lebanon
Maltese
MarcheneroPouter
MindianFantail
Mookee
OldDutchCapuchine
OldGermanOwl
OrientalFrill
OrientalRoller
ParlorRoller
PolishLynx
PomeranianPouter
PortugueseTumbler
PygmyPouter
RacingHomer
Runt
SaxonFairySwallow
SaxonMonk
SaxonPouter
Scandaroon
SchmalkaldenerMohrenkopf
Shakhsharli
ShowTypeHomer
SpanishBarb
Starling
SyrianDewlap
TemescheburgerSchecken
ThuringerCleanLeg
VoorburgShieldCropper
WestofEnglandTumbler

D)

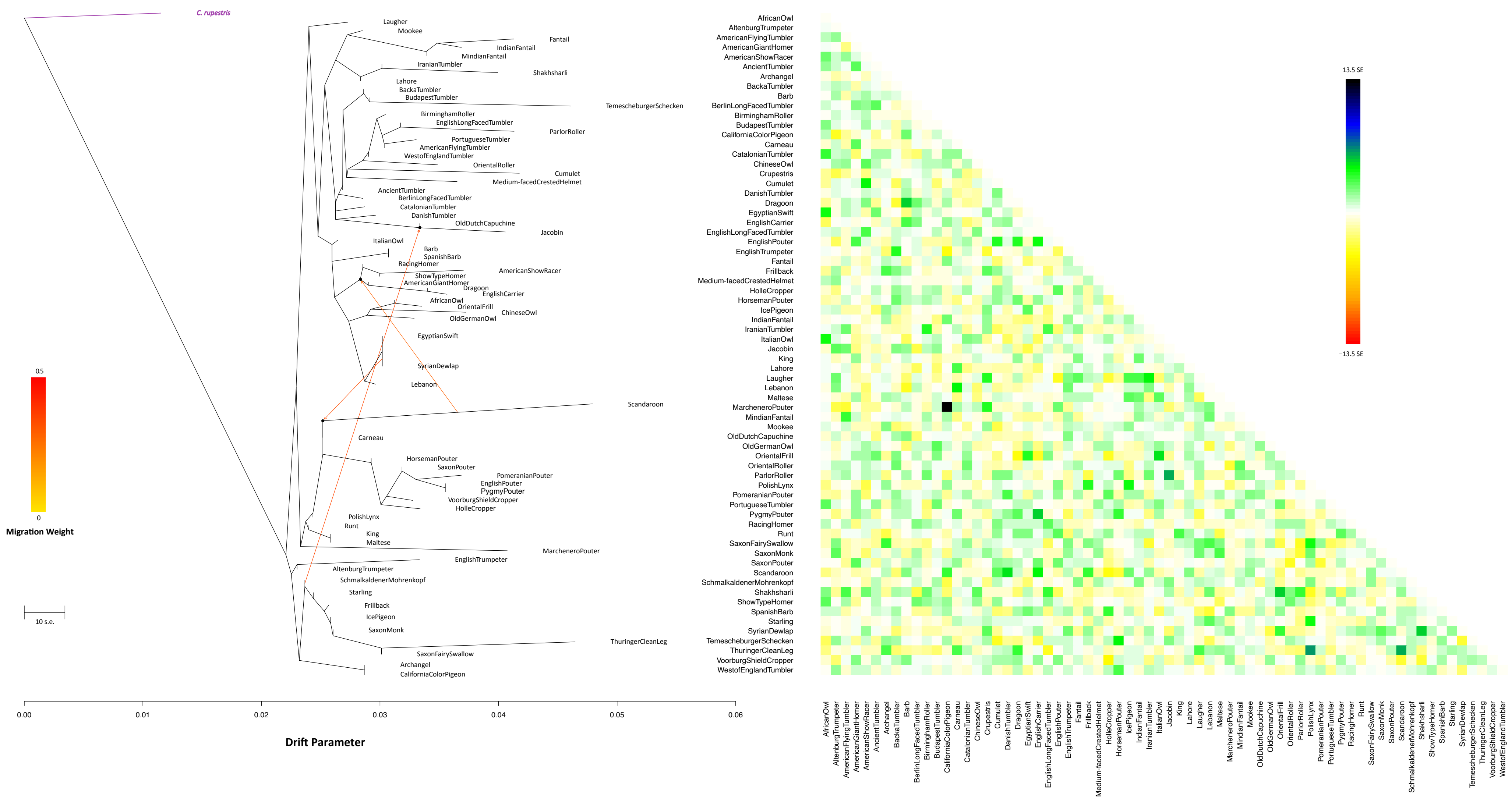
C. rupestris
Laugher
Mookee
Fantail
IndianFantail
MindianFantail
IranianTumbler
Shakhsharli
Lahore
BackaTumbler
BudapestTumbler
TemescheburgerSchecken
BirminghamRoller
EnglishLongFacedTumbler
ParlorRoller
PortugueseTumbler
AmericanFlyingTumbler
WestofEnglandTumbler
OrientalRoller
Cumulet
Medium-facedCrestedHelmet
AncientTumbler
BerlinLongFacedTumbler
CatalonianTumbler
DanishTumbler
OldDutchCapuchine
Jacobin
ItalianOwl
Barb
SpanishBarb
RacingHomer
AmericanShowRacer
ShowTypeHomer
AmericanGiantHomer
Dragoon
EnglishCarrier
AfricanOwl
OrientalFrill
ChineseOwl
OldGermanOwl
EgyptianSwift
SyrianDewlap
Lebanon
Scandaroon
Carneau
HorsemanPouter
SaxonPouter
PomeranianPouter
EnglishPouter
PygmyPouter
VoorburgShieldCropper
HolleCropper
PolishLynx
Runt
King
Maltese
MarcheneroPouter
EnglishTrumpeter
AltenburgTrumpeter
SchmalkaldenerMohrenkopf
Starling
Frillback
IcePigeon
SaxonMonk
ThuringerCleanLeg
SaxonFairySwallow
Archangel
CaliforniaColorPigeon
0.5
0
Migration Weight
10 s.e.
0.00
0.01
0.02
0.03
0.04
0.05
0.06
Drift Parameter
13.5 SE
-13.5 SE
AfricanOwl
AltenburgTrumpeter
AmericanFlyingTumbler
AmericanGiantHomer
AmericanShowRacer
AncientTumbler
Archangel
BackaTumbler
Barb
BerlinLongFacedTumbler
BirminghamRoller
BudapestTumbler
CaliforniaColorPigeon
Carneau
CatalonianTumbler
ChineseOwl
Crupestris
Cumulet
DanishTumbler
Dragoon
EgyptianSwift
EnglishCarrier
EnglishLongFacedTumbler
EnglishPouter
EnglishTrumpeter
Fantail
Frillback
Medium-facedCrestedHelmet
HolleCropper
HorsemanPouter
IcePigeon
IndianFantail
IranianTumbler
ItalianOwl
Jacobin
King
Lahore
Laugher
Lebanon
Maltese
MarcheneroPouter
MindianFantail
Mookee
OldDutchCapuchine
OldGermanOwl
OrientalFrill
OrientalRoller
ParlorRoller
PolishLynx
PomeranianPouter
PortugueseTumbler
PygmyPouter
RacingHomer
Runt
SaxonFairySwallow
SaxonMonk
SaxonPouter
Scandaroon
SchmalkaldenerMohrenkopf
Shakhsharli
ShowTypeHomer
SpanishBarb
Starling
SyrianDewlap
TemescheburgerSchecken
ThuringerCleanLeg
VoorburgShieldCropper
WestofEnglandTumbler

E)

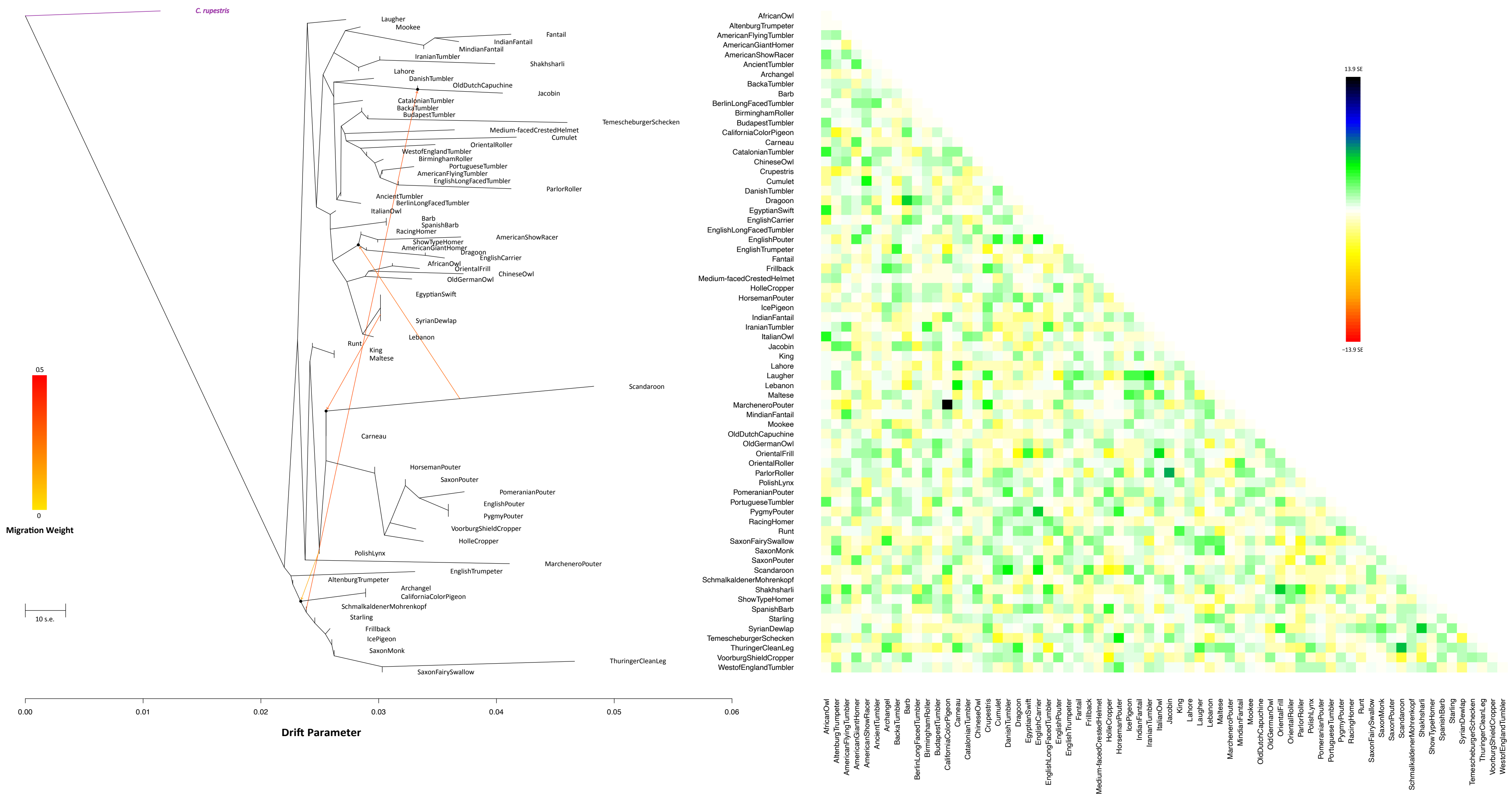
C. rupestris
Migration Weight
0.5
0
10 s.e.
Drift Parameter
0.00
0.01
0.02
0.03
0.04
0.05
0.06
13.9 SE
−13.9 SE
Laugher
Mookee
Fantail
IndianFantail
MindianFantail
IranianTumbler
Shakhsharli
Lahore
DanishTumbler
OldDutchCapuchine
Jacobin
CatalonianTumbler
BackaTumbler
BudapestTumbler
TemescheburgerSchecken
Medium-facedCrestedHelmet
Cumulet
OrientalRoller
WestofEnglandTumbler
BirminghamRoller
PortugueseTumbler
AmericanFlyingTumbler
EnglishLongFacedTumbler
ParlorRoller
AncientTumbler
BerlinLongFacedTumbler
ItalianOwl
Barb
SpanishBarb
RacingHomer
AmericanShowRacer
ShowTypeHomer
AmericanGiantHomer
Dragoon
EnglishCarrier
AfricanOwl
OrientalFrill
ChineseOwl
OldGermanOwl
EgyptianSwift
SyrianDewlap
Lebanon
Runt
King
Maltese
Scandaroon
Carneau
HorsemanPouter
SaxonPouter
PomeranianPouter
EnglishPouter
PygmyPouter
VoorburgShieldCropper
HolleCropper
PolishLynx
MarcheneroPouter
EnglishTrumpeter
AltenburgTrumpeter
Archangel
CaliforniaColorPigeon
SchmalkaldenerMohrenkopf
Starling
Frillback
IcePigeon
SaxonMonk
ThuringerCleanLeg
SaxonFairySwallow

F)

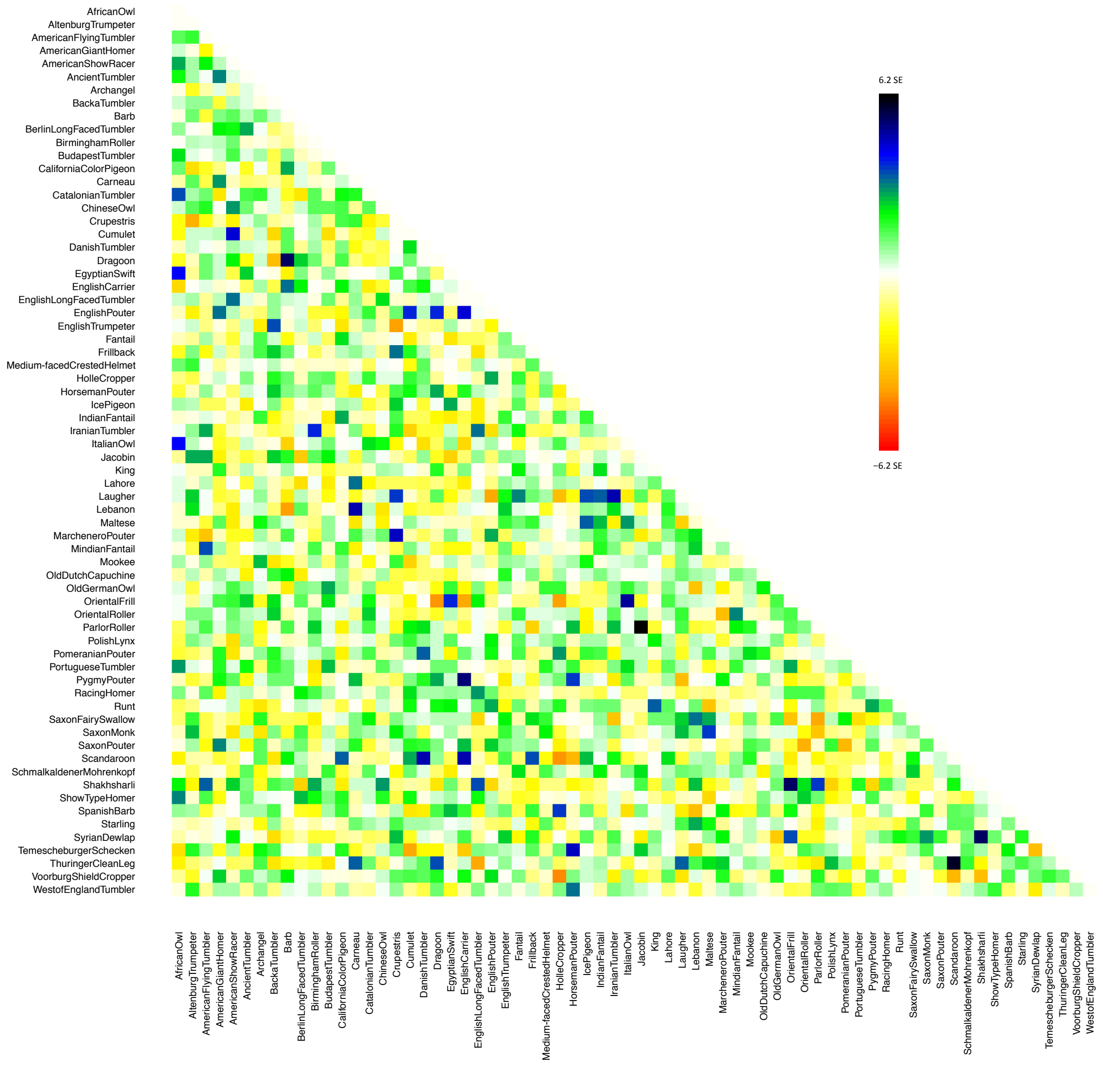

Supplementary Fig. 9. TreeMix phylogenies for pigeon breeds and model residuals. Model scenarios with (*A*) no migration edge, (*B*) one migration edge, (*C*) two migration edges, (*D*) three migration edges, (*E*) four migration edges, and (*F*) residuals of the five-migration model presented in Fig. 5. The outgroup is marked in purple in all phylogenetic plots.

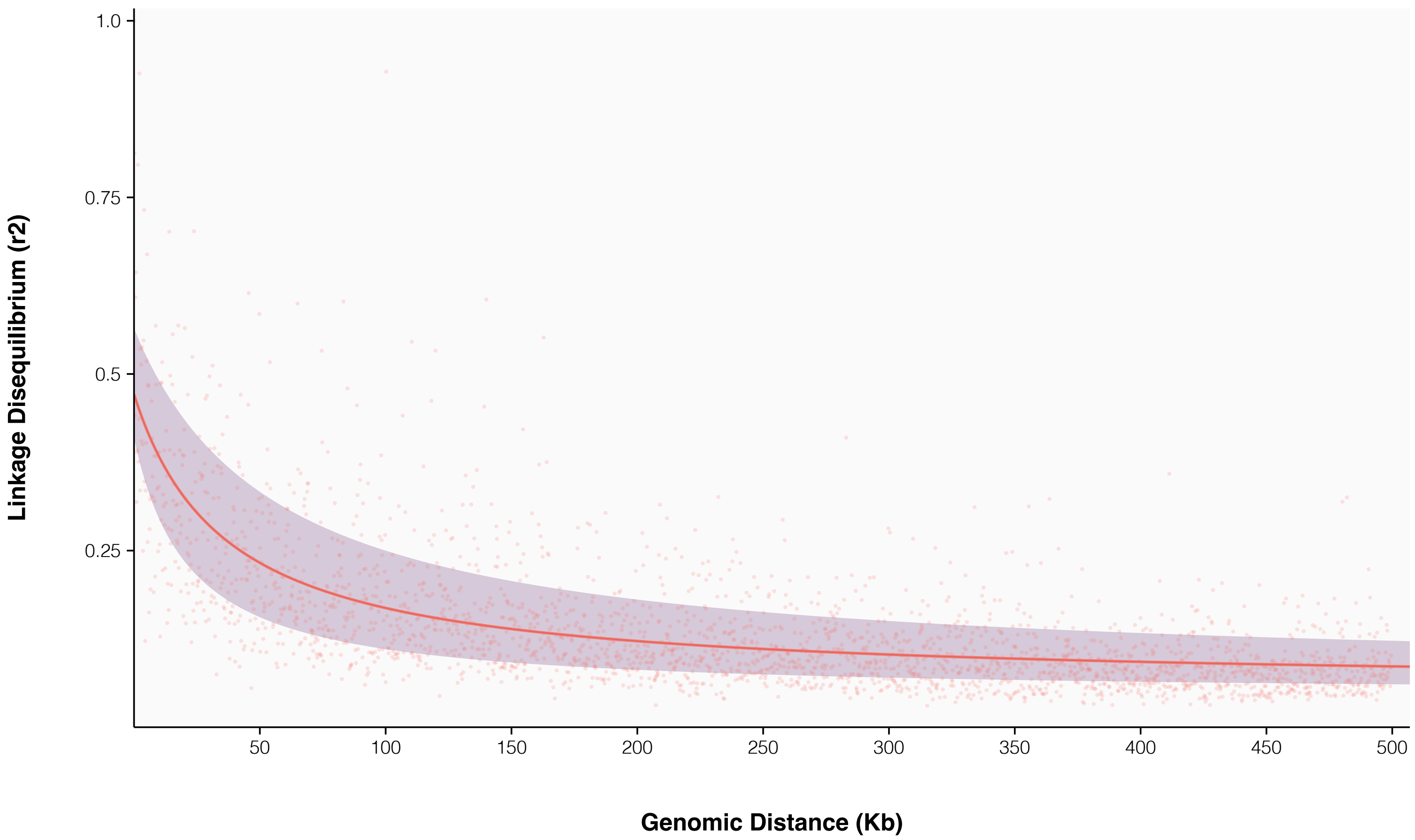

Supplementary Fig. 10. Linkage-disequilibrium (LD) across pigeon breeds. Decay of LD, as measured by r2, across physical distance. Pairwise LD was binned into 200-bp bins, and the 0.95 quantile used to infer the best-fitted curve (solid-line).

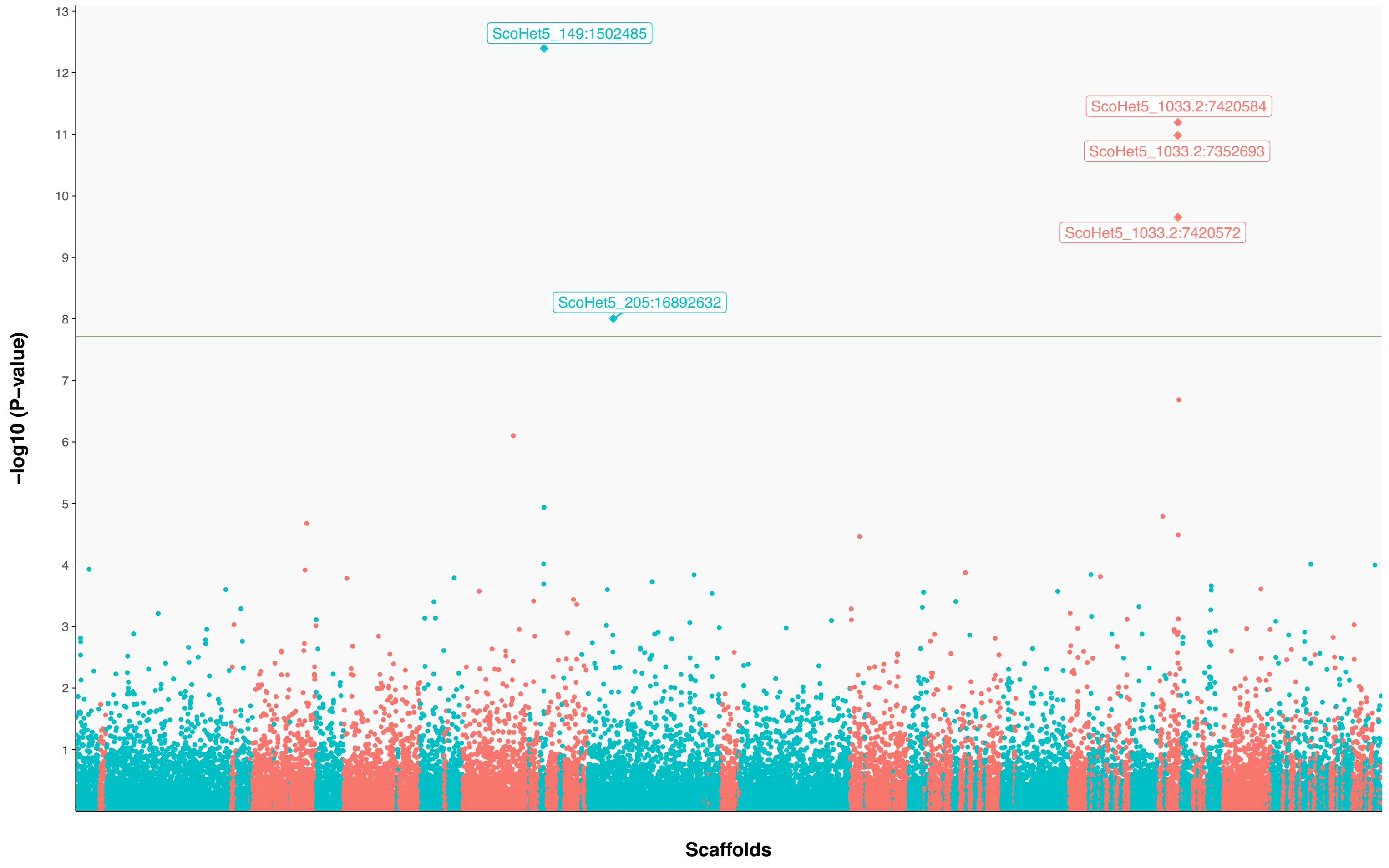
ScoHet5_149:1502485
ScoHet5_1033.2:7420584
ScoHet5_1033.2:7352693
ScoHet5_1033.2:7420572
ScoHet5_205:16892632
−log10 (P−value)
Scaffolds
13
12
11
10
9
8
7
6
5
4
3
2
1

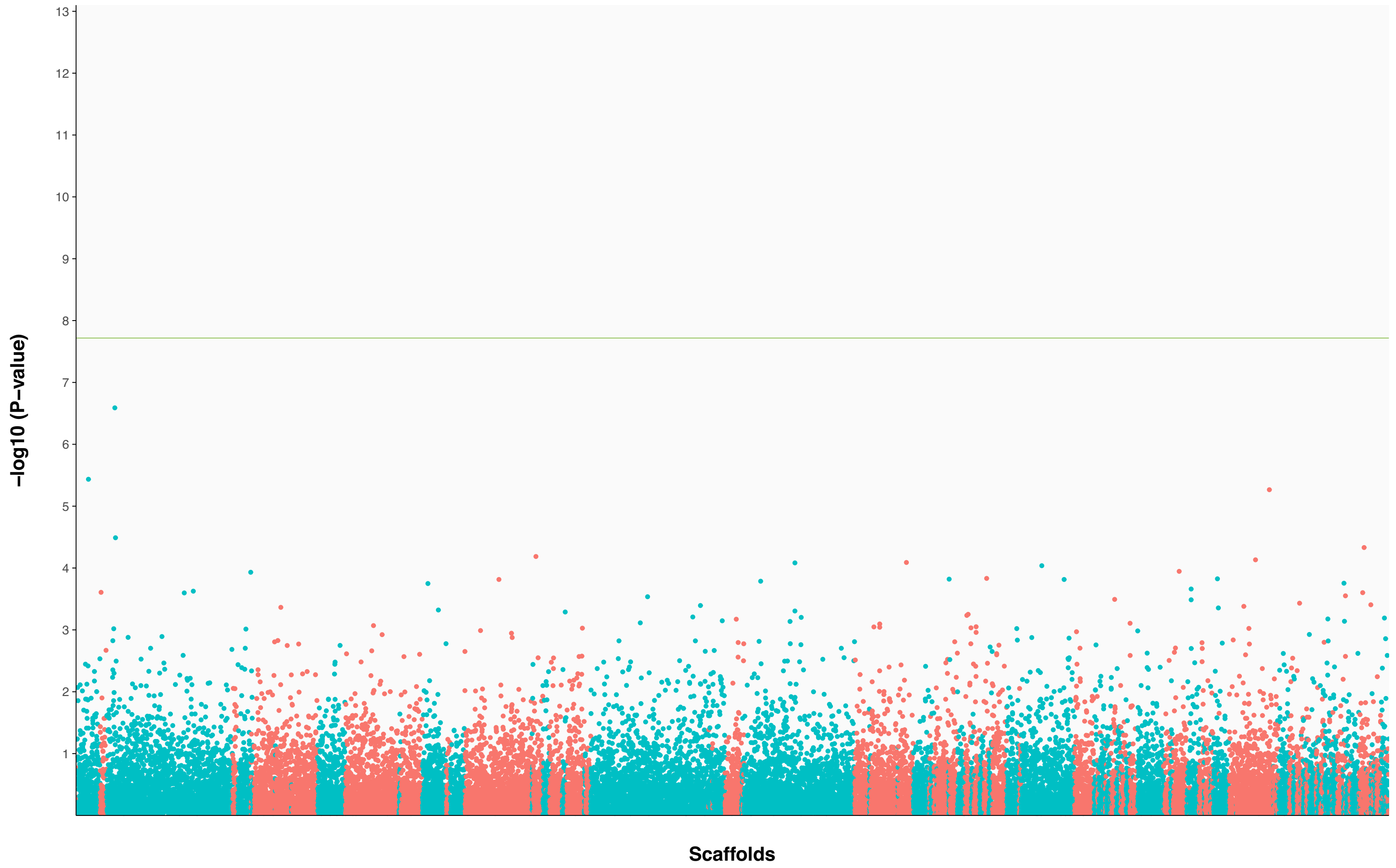

Supplementary Fig. 11. Manhattan plot for the GWAS analysis on the derived traits. (*A*) *FootFeathering* trait. (*B*) *Crest* trait. The green line indicates the permutation derived significance threshold for association (P < 1.91 X 10-8). The genomic coordinates of SNPs with significant associations to the phenotype are specified. The Y-axis shows the –log10-transformed two-tailed P-value of each SNP from the GWAS meta-analysis (of linear and logistic regression statistics), while the X-axis shows base-pair positions along the scaffolds.
